# Safety and immunogenicity of an oral, replicating adenovirus serotype 4 vector vaccine for H5N1 influenza: a randomised, double-blind, placebo-controlled, phase 1 study

**DOI:** 10.1016/S1473-3099(12)70345-6

**Published:** 2013-03

**Authors:** Marc Gurwith, Michael Lock, Eve M Taylor, Glenn Ishioka, Jeff Alexander, Tim Mayall, John E Ervin, Richard N Greenberg, Cynthia Strout, John J Treanor, Richard Webby, Peter F Wright

**Affiliations:** a PaxVax, San Diego, CA, USA; b The Center for Pharmaceutical Research, Kansas City, MO, USA; c University of Kentucky School of Medicine, Department of Medicine, Lexington, KY, USA; d Coastal Carolina Research Center, Mt Pleasant, SC, USA; e University of Rochester, Department of Medicine, Rochester, NY, USA; f St Jude Children's Hospital, Memphis, TN, USA; g Geisel Medical School at Dartmouth, Hanover, NH, USA

## Abstract

**Background:**

Replication-competent virus vector vaccines might have advantages compared with non-replicating vector vaccines. We tested the safety and immunogenicity of an oral adenovirus serotype 4 vector vaccine candidate (Ad4-H5-Vtn) expressing the haemagglutinin from an avian influenza A H5N1 virus.

**Methods:**

We did this phase 1 study at four sites in the USA. We used a computer-generated randomisation list (block size eight, stratified by site) to assign healthy volunteers aged 18–40 years to receive one of five doses of Ad4-H5-Vtn (10^7^ viral particles [VP], 10^8^ VP, 10^9^ VP, 10^10^ VP, 10^11^ VP) or placebo (3:1). Vaccine or placebo was given on three occasions, about 56 days apart. Participants, investigators, and study-site personnel were masked to assignment throughout the study. Subsequently, volunteers received a boost dose with 90 μg of an inactivated parenteral H5N1 vaccine. Primary immunogenicity endpoints were seroconversion by haemagglutination-inhibition (HAI), defined as a four-times rise compared with baseline titre, and HAI geometric mean titre (GMT). We solicited symptoms of reactogenicity daily for 7 days after each vaccination and recorded symptoms that persisted beyond 7 days as adverse events. Primary analysis was per protocol. This trial is registered with ClinicalTrials.gov, number NCT01006798.

**Findings:**

We enrolled 166 participants (125 vaccine; 41 placebo) between Oct 19, 2009, and Sept 9, 2010. HAI responses were low: 13 of 123 vaccinees (11%, 95% CI 6–17) and three of 41 placebo recipients (7%, 2–20) seroconverted. HAI GMT was 6 (95% CI 5–7) for vaccinees, and 5 (5–6) for placebo recipients. However, when inactivated H5N1 vaccine became available, one H5N1 boost was offered to all participants. In this substudy, HAI seroconversion occurred in 19 of 19 participants in the 10^11^ VP cohort (100%; 95% CI 82–100) and eight of 22 placebo recipients (36%; 17–59); 17 of 19 participants in the 10^11^ VP cohort (89%; 67–99) achieved seroprotection compared with four of 22 placebo recipients (18%; 5–40); GMT was 135 (89–205) with 10^11^ VP, compared with 13 (7–21) with placebo. The cumulative frequency of abdominal pain, diarrhoea, and nasal congestion after all three vaccinations was significantly higher in vaccinees than placebo recipients (21 [16·8%] of 125 *vs* one [2·4%] of 41, p=0·017; 24 [19·2%] of 125 *vs* two [4·9%] of 41, p=0·027; 41 [32·8%] of 125 *vs* six [14·6%] of 41, p=0·028; respectively). No serious treatment-related adverse events occurred.

**Interpretation:**

Oral Ad4 vector priming might enhance the efficacy of poorly immunogenic vaccines such as H5N1.

**Funding:**

Wellcome Trust Foundation, PaxVax.

## Introduction

Replication-competent (live) vaccine vectors have theoretical advantages compared with other vaccine approaches, because they combine the improved efficacy of live-attenuated vaccines with the safety of inactivated or subunit vaccines.^1^ There has been interest in replicating viral vectors as candidates for HIV^2^ or filovirus^3^ vaccines, and adenoviruses have been noted as having particular benefits as potential recombinant-vector vaccines for influenza^4^ and HIV.^5^ Only a few replicating vector viruses, other than flavivirus chimeric recombinants between related flaviviruses, have advanced to phase 1 studies.^6^, ^7^, ^8^, ^9^, ^10^ Candidate vaccine viruses of adenovirus serotypes 4 (Ad4) and 7 (Ad7), when bioengineered as replication-competent vectors, have the logistical and economic advantages of oral administration, are non-pathogenic by the oral route,^11^ remain fully replication competent, and, importantly, have substantial safety data having been given to more than 10 million US military recruits without notable adverse events.^11^ In 1991, a small phase 1 study assessed an Ad7-hepatitis B vector, but little replication occurred and no antibody response to the hepatitis B transgene was noted.^10^

We have developed Ad4 as a replicating vector virus, and as the first clinical assessment of this vector platform, did a large phase 1 study of an oral Ad4 vector-vaccine candidate expressing the haemagglutinin from an avian influenza A H5N1 virus. Our objectives were to assess safety of the vaccine, confirm that it replicates after oral administration, establish whether it expresses the haemagglutinin encoded by the transgene, and assess its ability to induce haemagglutinin-specific systemic and mucosal immune responses.

## Methods

### Study design and participants

This ascending dose, randomised, double-blind, placebo-controlled study was done at four sites (two university sites and two clinical research centres; appendix) in the USA. The study population was healthy men and non-pregnant women, aged 18–40 years, living with no more than two healthy, adult household contacts. We did not use baseline Ad4 serostatus as an entry criterion. Study participants and all their household contacts provided written informed consent and agreed to provide protocol-specified specimens for clinical laboratory, virological, and immunological assays. We obtained separate written informed consent from individuals who participated in a boost phase. The protocol was approved by institutional review boards and institutional biosafety committees at the participating centres.

### Randomisation and masking

Participants were enrolled in ascending dose cohorts and randomised independently within each (3:1) to either Ad4-H5-Vtn vaccine or placebo. Randomisation was stratified by site, with a masked block size of eight. At each site an independent, unmasked pharmacist dispensed either vaccine or placebo according to a computer-generated randomisation list. Participants, household contacts, investigators, study-site personnel, and monitors were masked to participants' treatment assignment throughout treatment and follow-up. Unmasked administration of the boost vaccination was offered to both vaccine and placebo recipients.

### Procedures

The vaccine, Ad4-H5-Vtn, is a recombinant, replication-competent Ad4, encoding full-length haemagglutinin from influenza A H5N1 virus (A/Vietnam/1194/2004). Its bioengineering and characteristics, including genetic stability, have been described elsewhere.^12^ The initial study design required about 96 volunteers to receive two vaccinations with one of three doses (10^7^ viral particles [VP], 10^8^ VP, or 10^9^ VP) of Ad4-H5-Vtn or placebo. For reference, the US military Ad4 vaccine, which is the Ad4 vector backbone, is currently dosed at no less than 10^4·5^ tissue culture infective dose_50_ (roughly equivalent to 10^7^ VP).^13^ After a blinded review showed very little antibody response within the 10^7^ VP, 10^8^ VP, or 10^9^ VP dose cohorts but no evidence of dose-related reactogenicity or toxic effects, the protocol was amended to add two higher dose groups, 10^10^ VP and 10^11^ VP, and administration of a third dose in all groups. The additional vaccination and the two higher dose groups were incorporated into the ascending dose design after approval from an independent data monitoring committee. After the inactivated parenteral subvirion H5N1 vaccine in the strategic national stockpile (A/Vietnam/1203/2004)^14^ became available, the protocol was further amended: all participants not lost to follow-up were offered a boost with this inactivated vaccine.

We solicited symptoms of reactogenicity daily for the 7 days after each vaccination (appendix); reactogenicity symptoms that persisted beyond 7 days were recorded as adverse events. Adverse events were recorded by site coordinators for at least 180 days after vaccination; clinical laboratory assessments were made at baseline and 7 days after each vaccination.

Immunological specimens were blood for Ad4 and H5N1 serology and cellular immune assays, and nasal and rectal wicks^15^, ^16^ for assessment of antibodies in mucosal secretions. We obtained specimens before each vaccination and at 28 days and 56 days after vaccination for Ad4 and H5N1 serology. We obtained cervical and rectal wicks^15^ from a subset of participants who consented to additional specimen collection. We assessed blood and rectal and throat swabs for Ad4-H5-Vtn by PCR on days 0, 7, 14, and 28 to assess shedding of vaccine virus or possible systemic spread; positive specimens were confirmed by Ad4 culture. We assessed potential transmission to household contacts by monitoring adverse events and by immunological and virological assessments: Ad4 and H5N1 serology on the day of each vaccination and 56 days later, and real-time (rt)PCR analysis of throat and rectal swabs 14 days after each vaccination. The rtPCR assay was specific for Ad4-H5-Vtn and would not be positive for wild-type Ad4. Any participant or household contact who developed signs or symptoms of illness compatible with clinical adenovirus infection was examined and appropriate clinical specimens (eg, urine or conjunctival swab), throat and rectal swabs, and blood were examined with rtPCR.

Serum haemagglutination-inhibition (HAI),^17^ microneutralisation^18^ for H5N1 (A/Vietnam/1194/2004) antibody, and neutralising antibody response to Ad4^19^ were assayed before and 28 days and 56 days after each vaccination. Additionally, H5 haemagglutinin-specific IgG and IgA ELISAs were done in plasma^20^ and in mucosal secretions.^13^ T-cell responses to haemagglutinin and Ad4 antigens were assessed in interferon-γ and interleukin-2 enzyme-linked immunosorbent spot (ELISPOT) assays^21^ with peripheral blood mononuclear cells stimulated with recombinant H5N1 haemagglutinin protein, for each of four pools of overlapping haemagglutinin peptides, or inactivated Ad4. Ad4-H5-Vtn virus in throat swabs, rectal swabs, and blood samples or clinical specimens was assessed by specific rtPCR (appendix).

### Statistical analysis

Two primary immunogenicity endpoints were prespecified: seroconversion by HAI, defined as a four-times rise compared with baseline titre, and HAI geometric mean titre (GMT). A sample size of 24 vaccine recipients per treatment group would give 76% power to detect a two-times difference in the occurrence of HAI seroconversion between any two treatment groups, with a two-sided Fisher's exact test with α=0·05 and assuming the occurrence in the better performing group was at least 80%. Primary analysis was per protocol, which required participants to have both a pre-vaccination and at least one post-vaccination HAI response.

We summarised categorical endpoints as percentages and 95% CIs, and compared them with Fisher's exact test. Continuous HAI, microneutralisation, and Ad4 neutralisation data were summarised as geometric means and CIs and compared with a *t* test on log-transformed data; ELISA and ELISPOT data were summarised as medians and IQRs and compared with the Wilcoxon signed-rank test. We used post-randomisation stratification to analyse the effect of baseline Ad4 seropositivity. Safety was summarised for all participants who received at least one vaccination with either Ad4-H5-Vtn or placebo; immunological analyses were done for subsets of participants who had results from before and after vaccination.

This trial is registered with ClinicalTrials.gov, number NCT01006798.

### Role of the funding source

The Wellcome Trust had no role in the study design or interpretation of data. The sponsor (PaxVax) designed the protocol in collaboration with participating investigators, and had overall responsibility for the conduct of the study. All authors vouch for accuracy and completeness of data. MG, ML, and ET had complete access to all data; all authors could access any of the data on request. Data were analysed by the biostatistician (ML), and the manuscript drafted by the first author (MG) with input from all authors.

## Results

**Figure 1:**
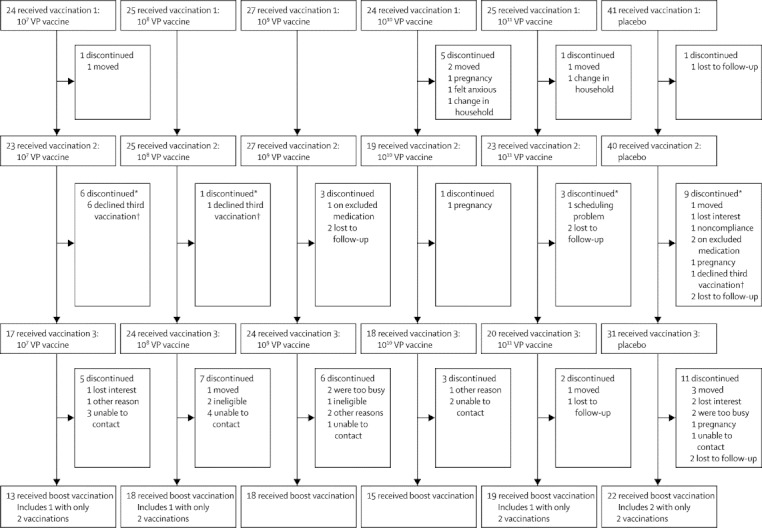
Trial profile Vaccination refers to oral administration of Ad4-H5-Vtn or oral placebo; boost vaccination refers to parenteral administration of subvirion inactivated H5N1 vaccine. Discontinuation means that a participant either dropped out of the study, or stopped vaccinations but continued follow-up. *Some participants who discontinued or missed vaccinations later received the boost vaccination. †Early entrants to the study were required to sign an additional consent form to receive the third vaccination but some declined to do so.

We enrolled 166 participants (125 vaccine recipients and 41 placebo recipients) between Oct 19, 2009, and Sept 9, 2010. Participants who were enrolled in the five vaccine cohorts received three vaccinations, separated by about 56 days each (figure 1). 105 (63%) participants (83 vaccinees and 22 placebo recipients) agreed to participate in the protein boost substudy and received 90 μg inactivated subvirion H5N1 3·5–15·5 months after their last dose of Ad4-H5-Vtn vaccine or placebo (figure 1). 88 household contacts, aged 18–61 years, took part in the study.

Ad4-H5-Vtn was not detected in any clinical specimen other than rectal swabs, with the exception of one vaccinee whose throat swab was positive for Ad4-H5-Vtn by PCR (and confirmed by culture) only on day 7. All his other specimens were negative. He was and remained entirely asymptomatic. Shedding of the Ad4-H5-Vtn was detected at day 14 in some vaccinees, particularly those who received lower doses (10^7^ VP–10^9^ VP). Thus, potentially, reactogenicity newly appearing during days 7–14 might have been missed, though we noted no pattern to suggest that vaccine-related adverse events occurred during this period. We identified no apparent relation of reactogenicity to dose for any specific solicited symptom or fever (Table 1, Table 2). For both vaccine and placebo groups, the highest occurrence of reactogenicity was reported after the first dose. Headache was the only reactogenicity symptom reported more frequently in vaccinees than placebo recipients after the first vaccination (p=0·046; table 1). The cumulative frequency of abdominal pain, diarrhoea, and nasal congestion after all three vaccinations was significantly higher in vaccinees than placebo recipients (21 [16·8%] of 125 *vs* one [2·4%] of 41, p=0·017; 24 [19·2%] of 125 *vs* two [4·9%] of 41, p=0·027; 41 [32·8%] of 125 *vs* six [14·6%] of 41, p=0·028; respectively; appendix). Symptoms were generally mild; none were dosage related or led to discontinuation of vaccine. After the H5N1 boost, systemic reactogenicity was much the same in Ad4-H5-Vtn and placebo recipients (appendix). Local reactogenicity after boost was more common in Ad4-H5-Vtn vaccinees: injection site pain (all mild) was reported by 17 (20·5%) of 83 vaccinees and none of 22 placebo recipients (p=0·020). However, the occurrence of tenderness at the injection site was similar between groups: 24 (28·9%) of 83 vaccinees and five (22·7%) of 22 placebo recipients. Severe tenderness was reported in three vaccinees and no placebo recipients (appendix).

**Table 1:** Signs and symptoms of reactogenicity and highest severity level reported after first dose

		**Ad4-H5-Vtn vaccine recipients**						**Placebo (n=41)**
		10^7^ VP (n=24)	10^8^ VP (n=25)	10^9^ VP (n=27)	10^10^ VP (n=24)	10^11^ VP (n=25)	All doses combined (n=125)	
**Abdominal pain**								
Any		2 (8·3%)	3 (12·0%)	4 (14·8%)	3 (12·5%)	1 (4·0%)	13 (10·4%)	1 (2·4%)
	Mild	1 (4·2%)	3 (12·0%)	2 (7·4%)	3 (12·5%)	1 (4·0%)	10 (8·0%)	1 (2·4%)
	Moderate	1 (4·2%)	0	2 (7·4%)	0	0	3 (2·4%)	0
	Severe	0	0	0	0	0	0	0
**Diarrhoea**								
Any		2 (8·3%)	3 (12·0%)	3 (11·1%)	5 (20·8%)	4 (16·0%)	17 (13·6%)	2 (4·9%)
	Mild	2 (8·3%)	3 (12·0%)	0	3 (12·5%)	4 (16·0%)	12 (9·6%)	2 (4·9%)
	Moderate	0	0	3 (11·1%)	2 (8·3%)	0	5 (4·0%)	0
	Severe	0	0	0	0	0	0	0
**Nausea or vomiting**								
Any		1 (4·2%)	1 (4·0%)	3 (11·1%)	1 (4·2%)	3 (12·0%)	9 (7·2%)	1 (2·4%)
	Mild	1 (4·2%)	1 (4·0%)	1 (3·7%)	1 (4·2%)	3 (12·0%)	7 (5·6%)	1 (2·4%)
	Moderate	0	0	2 (7·4%)	0	0	2 (1·6%)	0
	Severe	0	0	0	0	0	0	0
**Chills**								
Any		0	1 (4·0%)	0	1 (4·2%)	0	2 (1·6%)	0
	Mild	0	1 (4·0%)	0	0	0	1 (0·8%)	0
	Moderate	0	0	0	1 (4·2%)	0	1 (0·8%)	0
	Severe	0	0	0	0	0	0	0
**Muscle or body aches**								
Any		1 (4·2%)	1 (4·0%)	4 (14·8%)	2 (8·3%)	3 (12·0%)	11 (8·8%)	1 (2·4%)
	Mild	0	1 (4·0%)	2 (7·4%)	1 (4·2%)	3 (12·0%)	7 (5·6%)	1 (2·4%)
	Moderate	1 (4·2%)	0	2 (7·4%)	1 (4·2%)	0	4 (3·2%)	0
	Severe	0	0	0	0	0	0	0
**Joint pain**								
Any		1 (4·2%)	0	2 (7·4%)	1 (4·2%)	1 (4·0%)	5 (4%)	1 (2·4%)
	Mild	1 (4·2%)	0	1 (3·7%)	0	1 (4·0%)	3 (2·4%)	0
	Moderate	0	0	1 (3·7%)	1 (4·2%)	0	2 (1·6%)	1 (2·4%)
	Severe	0	0	0	0	0	0	0
**Tiredness**								
Any		6 (25·0%)	7 (28·0%)	6 (22·2%)	5 (20·8%)	4 (16·0%)	28 (22·4%)	5 (12·2%)
	Mild	2 (8·3%)	4 (16·0%)	1 (3·7%)	2 (8·3%)	4 (16·0%)	13 (10·4%)	2 (4·9%)
	Moderate	4 (16·7%)	3 (12·0%)	4 (14·8%)	3 (12·5%)	0	14 (11·2%)	3 (7·3%)
	Severe	0	0	1 (3·7%)	0	0	1 (0·8%)	0
**Headache**								
Any		7 (29·2%)	5 (20·0%)	10 (37·0%)	7 (29·2%)	5 (20·0%)	34 (27·2%)*	6 (14·6%)
	Mild	4 (16·7%)	4 (16·0%)	6 (22·2%)	4 (16·7%)	4 (16·0%)	22 (17·6%)	3 (7·3%)
	Moderate	3 (12·5%)	1 (4·0%)	4 (14·8%)	3 (12·5%)	1 (4·0%)	12 (9·6%)	3 (7·3%)
	Severe	0	0	0	0	0	0	0
**Nasal congestion or runny nose**								
Any		5 (20·8%)	6 (24·0%)	4 (14·8%)	2 (8·3%)	2 (8·0%)	19 (15·2%)	3 (7·3%)
	Mild	2 (8·3%)	4 (16·0%)	3 (11·1%)	1 (4·2%)	2 (8·0%)	12 (9·6%)	2 (4·9%)
	Moderate	3 (12·5%)	2 (8·0%)	1 (3·7%)	1 (4·2%)	0	7 (5·6%)	1 (2·4%)
	Severe	0	0	0	0	0	0	0
**Sore throat**								
Any		2 (8·3%)	3 (12·0%)	1 (3·7%)	6 (25·0%)	1 (4·0%)	13 (10·4%)	4 (9·8%)
	Mild	1 (4·2%)	3 (12·0%)	0	4 (16·7%)	1 (4·0%)	9 (7·2%)	3 (7·3%)
	Moderate	1 (4·2%)	0	1 (3·7%)	2 (8·3%)	0	4 (3·2%)	1 (2·4%)
	Severe	0	0	0	0	0	0	0
**Cough**								
Any		2 (8·3%)	2 (8·0%)	1 (3·7%)	3 (12·5%)	0	8 (6·4%)	1 (2·4%)
	Mild	2 (8·3%)	2 (8·0%)	0	3 (12·5%)	0	7 (5·6%)	1 (2·4%)
	Moderate	0	0	1 (3·7%)	0	0	1 (0·8%)	0
	Severe	0	0	0	0	0	0	0
**Shortness of breath**								
Any		1 (4·2%)	0	1 (3·7%)	0	0	2 (1·6%)	0
	Mild	0	0	0	0	0	0	0
	Moderate	1 (4·2%)	0	1 (3·7%)	0	0	2 (1·6%)	0
	Severe	0	0	0	0	0	0	0
**Fever**								
Any		0	0	0	0	0	0	0
	Mild	0	0	0	0	0	0	0
	Moderate	0	0	0	0	0	0	0
	Severe	0	0	0	0	0	0	0

Data are n (%) who reported that symptom at least once in the 7 days after the first dose.

* The group comprised of all Ad4-H5-Vtn recipients reported significantly more headaches than did placebo recipients (p=0·046).

**Table 2:** Participants reporting reactogenicity signs or symptoms

		**Ad4-H5-Vtn vaccine recipients**						**Placebo (n=41)**
		10^7^ VP (n=24)	10^8^ VP (n=25)	10^9^ VP (n=27)	10^10^ VP (n=24)	10^11^ VP (n=25)	All doses combined (n=125)	
**Abdominal pain**								
Vaccination number								
	1	2/24 (8%)	3/25 (12%)	4/27 (15%)	3/24 (13%)	1/25 (4%)	13/125 (10%)	1/41 (2%)
	2	2/23 (9%)	0/25	1/27 (4%)	2/19 (11%)	0/23	5/117 (4%)	0/40
	3	1/17 (6%)	1/24 (4%)	0/24	0/18	2/20 (10%)	4/103 (4%)	0/31
**Diarrhoea**								
Vaccination number								
	1	2/24 (8%)	3/25 (12%)	3/27 (11%)	5/24 (21%)	4/25 (16%)	17/125 (14%)	2/41 (5%)
	2	2/23 (9%)	1/25 (4%)	1/27 (4%)	2/19 (11%)	1/23 (4%)	7/117 (6%)	0/40
	3	1/17 (6%)	1/24 (4%)	0/24	0/18	1/20 (5%)	3/103 (3%)	0/31
**Nausea or vomiting**								
Vaccination number								
	1	1/24 (4%)	1/25 (4%)	3/27 (11%)	1/24 (4%)	3/25 (12%)	9/125 (7%)	1/41 (2%)
	2	1/23 (4%)	2/25 (8%)	1/27 (4%)	0/19	1/23 (4%)	5/117 (4%)	1/40 (3%)
	3	0/17	1/24 (4%)	0/24	0/18	2/20 (10%)	3/103 (3%)	1/31 (3%)
**Chills**								
Vaccination number								
	1	0/24	1/25 (4%)	0/27	1/24 (4%)	0/25	2/125 (2%)	0/41
	2	1/23 (4%)	2/25 (8%)	1/27 (4%)	0/19	0/23	4/117 (3%)	0/40
	3	0/17	1/24 (4%)	0/24	1/18 (6%)	0/20	2/103 (2%)	0/31
**Muscle or body aches**								
Vaccination number								
	1	1/24 (4%)	1/25 (4%)	4/27 (15%)	2/24 (8%)	3/25 (12%)	11/125 (9%)	1/41 (2%)
	2	1/23 (4%)	2/25 (8%)	2/27 (7%)	1/19 (5%)	0/23	6/117 (5%)	1/40 (3%)
	3	0/17	2/24 (8%)	0/24	2/18 (11%)	1/20 (5%)	5/103 (5%)	1/31 (3%)
**Joint pain**								
Vaccination number								
	1	1/24 (4%)	0/25	2/27 (7%)	1/24 (4%)	1/25 (4%)	5/125 (4%)	1/41 (2%)
	2	0/23	0/25	0/27	0/19	0/23	0/117	0/40
	3	0/17	0/24	0/24	1/18 (6%)	0/20	1/103 (1%)	0/31
**Tiredness**								
Vaccination number								
	1	6/24 (25%)	7/25 (28%)	6/27 (22%)	5/24 (21%)	4/25 (16%)	28/125 (22%)	5/41 (12%)
	2	2/23 (9%)	0/25	3/27 (11%)	2/19 (11%)	0/23	7/117 (6%)	0/40
	3	2/17 (12%)	0/24	1/24 (4%)	2/18 (11%)	1/20 (5%)	6/103 (6%)	2/31 (6%)
**Headache**								
Vaccination number								
	1	7/24 (29%)	5/25 (20%)	10/27 (37%)	7/24 (29%)	5/25 (20%)	34/125 (27%)	6/41 (15%)
	2	2/23 (9%)	2/25 (8%)	6/27 (22%)	4/19 (21%)	2/23 (9%)	16/117 (14%)	5/40 (13%)
	3	4/17 (24%)	3/24 (13%)	3/24 (13%)	3/18 (17%)	1/20 (5%)	14/103 (14%)	1/31 (3%)
**Nasal congestion or runny nose**								
Vaccination number								
	1	5/24 (21%)	6/25 (24%)	4/27 (15%)	2/24 (8%)	2/25 (8%)	19/125 (15%)	3/41 (7%)
	2	6/23 (26%)	2/25 (8%)	7/27 (26%)	2/19 (11%)	2/23 (9%)	19/117 (16%)	3/40 (8%)
	3	3/17 (18%)	3/24 (13%)	2/24 (8%)	0/18	1/20 (5%)	9/103 (9%)	1/31 (3%)
**Sore throat**								
Vaccination number								
	1	2/24 (8%)	3/25 (12%)	1/27 (4%)	6/24 (25%)	1/25 (4%)	13/125 (10%)	4/41 (10%)
	2	2/23 (9%)	0/25	4/27 (15%)	3/19 (16%)	1/23 (4%)	10/117 (9%)	1/40 (3%)
	3	3/17 (18%)	1/24 (4%)	2/24 (8%)	1/18 (6%)	1/20 (5%)	8/103 (8%)	0/31
**Cough**								
Vaccination number								
	1	2/24 (8%)	2/25 (8%)	1/27 (4%)	3/24 (13%)	0/25	8/125 (6%)	1/41 (2%)
	2	2/23 (9%)	1/25 (4%)	4/27 (15%)	0/19	0/23	7/117 (6%)	2/40 (5%)
	3	3/17 (18%)	1/24 (4%)	1/24 (4%)	0/18	1/20 (5%)	6/103 (6%)	0/31
**Shortness of breath**								
Vaccination number								
	1	1/24 (4%)	0/25	1/27 (4%)	0/24	0/25	2/125 (2%)	0/41
	2	0/23	1/25 (4%)	0/27	0/19	0/23	1/117 (1%)	0/40
	3	1/17 (6%)	0/24	0/24	0/18	0/20	1/103 (1%)	1/31 (3%)
**Fever**								
Vaccination number								
	1	0/24	0/25	0/27	0/24	0/25	0/125	0/41
	2	0/23	0/25	0/27	1/19 (5%)	0/23	1/117 (1%)	0/40
	3	0/17	1/24 (4%)	0/24	0/18	0/20	1/103 (1%)	0/31

Data are n/N (%).

No discontinuations for toxic effects occurred (figure 1). Three participants had serious adverse events: hallucinations 6 months after the last vaccination and norovirus-associated gastroenteritis in the vaccine group, and pancreatitis in the placebo group. None were judged to be related to the study.

Ad4-H5-Vtn was not detected by PCR analysis of any clinical sample or rectal or throat swabs from any household contacts. However, two (3·4%) of 58 household contacts of vaccinees, and one (3·3%) of 30 household contacts of placebo recipients had asymptomatic seroconversions to Ad4.

**Figure 2:**
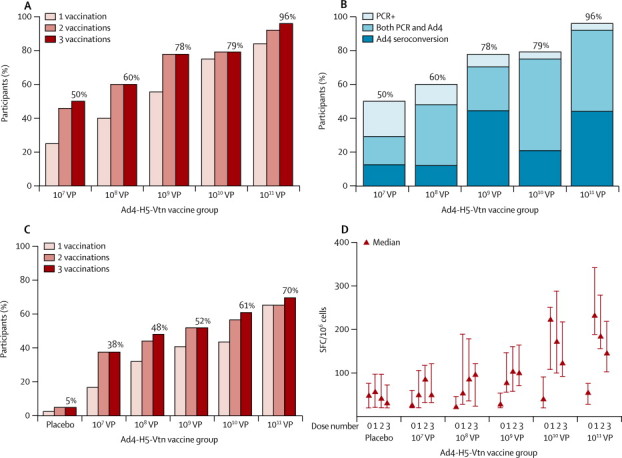
Vaccine take and cellular immune response by vaccination and by median number of ELISPOT forming cells Cumulative vaccine take (A) defined as Ad4 seroconversion or a PCR-positive rectal swab at 7 days or 14 days after vaccination, or both; cumulative percentage of participants with at least one take after one, two, and three Ad4-H5-Vtn vaccinations. Vaccine take components (B) partitioned to show proportion of participants who had PCR-positive rectal swab, Ad4 seroconversion, or both after any vaccination; data not available for placebo recipients, who were not assessed for shedding of vaccine virus. Cumulative percentage of participants with an ELISPOT interferon-γ response (C), defined as 80 or more spot forming cells (SFC) and four-times greater than baseline SFC, after one, two, and three vaccinations. Median number of ELISPOT SFC/10^6^ cells at baseline and after each vaccination (D); error bars show IQR. ELISPOT=enzyme-linked immunosorbent spot.

Replication of the Ad4-H5-Vtn vaccine virus or so-called vaccine take (Ad4-H5-Vtn detected in rectal swabs or seroconversion to Ad4, or both) increased with increasing dose (figure 2). A dose-response association was especially apparent after the first vaccination, for which occurrence of take ranged from 25% (six of 24) with 10^7^ VP to 84% (21 of 25) with 10^11^ VP. Overall, 57 (46%) of 125 of vaccinees had PCR-positive rectal swabs, with or without associated Ad4 seroconversion. We identified PCR-positive swabs in roughly equal numbers in the lower dose cohorts (10^7^ VP–10^9^ VP) at both 7 days and 14 days and in the high-dose cohorts (10^10^ VP and 10^11^ VP) almost exclusively at 7 days (appendix), consistent with a shorter incubation period in participants receiving high doses. Of the 57 vaccinees with at least one PCR-positive rectal swab, an Ad4-H5-Vtn virus was detected by culture in 38 (67%). We identified no relation between dose and culture positivity. Occurrence of Ad4 seroconversion ranged from 29% to 96% and GMTs from 4 to 130 (figure 2B).

Although cumulative take after the second vaccination showed much the same dose association as for the first vaccination, the greatest effect of additional vaccinations was in the lower dose cohorts (10^7^ VP–10^9^ VP), in which participants without a take after the first or second administration subsequently had a take (figure 2).

Interferon-γ T-cell response to recombinant H5 haemagglutinin protein or peptide pools ranged from 38% (nine of 24 vaccinees) with 10^7^ VP to 70% (16 of 23 vaccines) with 10^11^ VP, compared with only 5% (two of 41) of placebo recipients (figure 2). Interleukin-2 responses were much the same (appendix). Vaccinees in the 10^10^ VP and 10^11^ VP groups showed the largest overall response after the initial vaccination. Median response for interferon γ was 232 spots per 10^6^ cells with 10^11^ VP compared with 57 spots per 10^6^ cells with placebo (p<0·0001). Median response for interleukin 2 was 345 spots per 10^6^ cells with 10^11^ VP compared with 111 spots per 10^6^ for placebo (p<0·0001). In specimens with sufficient blood remaining, pilot studies analysing intracellular cytokine production or CD4 and CD8 depleted ELISPOT assays showed that responses were predominantly induced by CD4-positive T cells (data not shown).

By contrast with the cellular response, haemagglutinin-specific antibody responses before boosting were scarce and of low titres (Table 3, Table 4). HAI seroconversion ranged from 4% to 19% among the vaccine cohorts, compared with 7% among placebo recipients (Table 3, Table 4). Only five vaccinees and no placebo recipients achieved seroprotection (HAI titre ≥40). However, after one boost with inactivated H5N1 vaccine, Ad4-H5-Vtn recipients had significantly higher occurrence of seroconversion than did placebo recipients: 80% for HAI and 67% for microneutralisation in vaccinees, compared with 36% and 33%, respectively, in placebo recipients (Table 3, Table 4; p=0·0003 for HAI, p=0·006 for microneutralisation). Across the five vaccine cohorts, occurrence of HAI seroconversion ranged from 67% to 100%; microneutralisation seroconversion ranged from 56% to 80% (Table 3, Table 4). Post-boost HAI GMT for all vaccine cohorts was 56 (range 28–135), compared with 13 for the placebo group (Table 3, Table 4; p<0·0001 for all groups *vs* placebo; GMT 10·7, 95% CI 5·5–20·9). Seroprotection ranged from 61% to 89% in vaccine groups, compared with 18% for placebo (Table 3, Table 4). We identified evidence of heterologous (cross-clade) HAI response with the homologous prime and homologous boost. In a pilot substudy, all participants with post-boost HAI titres of 80 or more were assessed for cross-clade responses. 15 of the 16 vaccinees (and one of two placebo recipients) with post-boost HAI titres of 80 or more to A/Vietnam/2004 (clade 1) had seroprotective titres (HAI ≥40) to A/Dk/Hunan/2002 (a clade 2.1 H5 virus), suggesting that Ad4-H5 clade 1 prime, followed by the subvirion clade 1 boost immunisation, induced sufficient heterologous antibody response to a clade 2.1 H5 virus to predict protection against this subtype.

**Table 3:** Cumulative seroconverted by HAI and GMT after each vaccination

			**Ad4-H5-Vtn vaccine recipients**						**Placebo**
			10^7^ VP	10^8^ VP	10^9^ VP	10^10^ VP	10^11^ VP	All doses combined	
Pre-boost vaccinations			n=24	n=25	n=27	n=23	n=24	n=123	n=41
	Day 56 (one vaccination)								
		% seroconverted (95% CI)	0% (0–14)	4% (0–20)	7% (1–24)	0% (0–15)	4% (0–21)	3% (1–8)	5% (1–17)
		GMT (95% CI)	5 (NC)	6 (5–7)	6 (5–6)	5 (5–6)	6 (5–8)	6 (5–6)	5 (5–5)
	Day 112 (two vaccinations)								
		% seroconverted (95% CI)	4% (0–21)	8% (1–26)	15% (4–34)	4% (0–22)	13% (3–32)	9% (5–15)	5% (1–17)
		GMT (95% CI)	5 (5–6)	7 (5–10)	6 (5–7)	6 (5–7)	6 (5–8)	6 (6–7)	5 (NC)
	Day 140 (three vaccinations)								
		% seroconverted (95% CI)	4% (0–21)	12% (3–31)	19% (6–38)	4% (0–22)	13% (3–32)	11% (6–17)	7% (2–20)
		GMT (95% CI)	5 (5–6)	8 (5–12)	6 (5–7)	5 (5–6)	7 (5–9)	6 (5–7)	5 (5–6)
Boost vaccination			n=13	n=18	n=18	n=15	n=19	n=83	n=22
	% seroconverted (95% CI)		69% (39–91)	78% (52–94)	67% (41–87)	80% (52–96)	100% (82–100)	80% (69–88)*	36% (17–59)
	% seroprotected (95% CI)		62% (32–86)	67% (41–87)	61% (36–83)	80% (52–96)	89% (67–99)	72% (61–82)†	18% (5–40)
	GMT (95% CI)		28 (12–64)	48 (24–94)	34 (16–74)	77 (38–153)	135 (89–205)	56 (42–76)†	13 (7–21)

For HAI, pre-boost seroconversion is a four-times or greater rise compared with baseline. Post-boost seroconversion is a four-times or greater rise compared with the titre obtained just before boost vaccination. Seroprotection is a titre ≥40. Pre-boost percentages are cumulative—eg, a participant who seroconverted by day 56 is counted as seroconverted at days 112 and 140 also. Pre-boost percentages are calculated from participants (n) who had a baseline result and at least one post-vaccination result. Post-boost percentages are calculated from participants (n) who had both pre-boost and post-boost results. Missing post-vaccination results are counted as negative responses. GMTs are calculated from all data available at the indicated timepoint. HAI=haemagglutination-inhibition. GMT=geometric mean titre. NC=not calculable.

* p=0·0003.

† p<0·0001.

**Table 4:** Cumulative seroconverted by H5 haemagglutinin microneutralisation and GMT after each vaccination

			**Ad4-H5-Vtn vaccine recipients**						**Placebo**
			10^7^ VP	10^8^ VP	10^9^ VP	10^10^ VP	10^11^ VP	All doses combined	
Pre-boost vaccinations			n=24	n=25	n=27	n=21	n=24	n=121	n=40
	Day 56 (one vaccination)								
		% seroconverted (95% CI)	4% (0–21)	4% (0–20)	0% (0–13)	5% (0–24)	0% (0–14)	2% (1– 7)	3% (0–13)
		GMT (95% CI)	5 (NC)	7 (6–9)	7 (6–9)	6 (5–8)	6 (5–6)	6 (6–7)	6 (5–7)
	Day 112 (two vaccinations)								
		% seroconverted (95% CI)	4% (0–21)	4% (0–20)	7% (1–24)	5% (0–24)	8% (1–27)	6% (2– 12)	3% (0– 13)
		GMT (95% CI)	5 (5–6)	6 (5–7)	9 (7–11)	6 (5–6)	7 (5–8)	6 (6–7)	6 (5–6)
	Day 140 (three vaccinations)								
		% seroconverted (95% CI)	4% (0–21)	4% (0–20)	7% (1–24)	5% (0–24)	13% (3–32)	7% (3– 13)	3% (0–13)
		GMT (95% CI)	5 (NC)	6 (5–8)	9 (7–11)	6 (5–7)	7 (6–9)	7 (6–7)	6 (5–7)
Boost vaccination			n=13	n=18	n=18	n=15	n=18	n=82	n=21
	% seroconverted (95% CI)		62% (32–86)	67% (41–87)	56% (31–78)	80% (52–96)	72% (47–90)	67% (56–77)*	33% (15–57)
	% seroprotected (95% CI)		54% (25–81)	50% (26–74)	56% (31–78)	60% (32–84)	50% (26–74)	54% (42–65)†	14% (3–35)
	GMT (95% CI)		48 (22–103)	46 (22–95)	45 (18–112)	97 (38–251)	42 (26–69)	52 (37–72)‡	14 (9–22)

For H5 haemagglutinin microneutralisation, pre-boost seroconversion is a four-times or greater rise compared with baseline. Post-boost seroconversion is a four-times or greater rise compared with the titre obtained just before boost vaccination. Seroprotection is a titre ≥40. Pre-boost percentages are cumulative—eg, a participant who seroconverted by day 56 is counted as seroconverted at days 112 and 140 also. Pre-boost percentages are calculated from participants (n) who had a baseline result and at least one post-vaccination result. Post-boost percentages are calculated from participants (n) who had both pre-boost and post-boost results. Missing post-vaccination results are counted as negative responses. GMTs are calculated from all data available at the indicated timepoint. HAI=haemagglutination-inhibition. GMT=geometric mean titre. NC=not calculable.

* p=0·006.

† p=0·0007.

‡ p=0·0001.

Plasma IgA ELISA seroresponse generally mirrored the HAI and microneutralisation response before boost, but the IgG ELISA response was more robust. In the highest dose cohort, 12 (50%) of 24 were IgG seropositive, compared with two (5%) of 39 in the placebo group. After-boost responses were strong, with 100% responding to IgG in the highest dose cohort; 50% (11 of 22) of placebo recipients were IgG seropositive after boost. Similar to serum findings, occurrence of IgG and IgA response in before-boost cervical, rectal, and nasal mucosal samples were low (data not shown). After boost, we noted that IgG responses in nasal mucosal samples were generally higher in vaccinees than in the placebo group (cervical and rectal specimens were not obtained post-boost; appendix).

**Figure 3:**
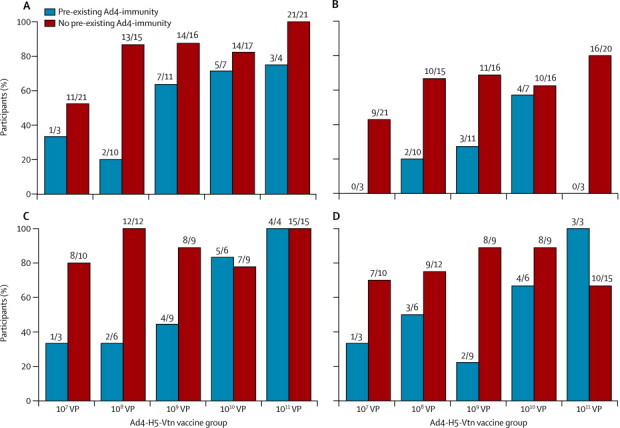
Effect of pre-existing Ad4 immunity on occurrence of take, cellular immune response before boost, and seroconversion by HAI and H5 haemagglutininin microneutralisation after boost For vaccine take (A), defined as Ad4 seroconversion or a PCR-positive rectal swab at 7 or 14 days after vaccination, or both, bar height represents the cumulative percentage of participants with a take at any time before study completion or receipt of boost vaccination. Analogous percentages for ELISPOT interferon-γ response before boost, defined as 80 or more spot forming cells (SFC) and four-times greater than baseline SFC, are shown (B). HAI seroconversion after boost (C) and H5 haemagglutinin microneutralisation (D) required a four-times rise compared with the last result before boost. The number of responders and the number of participants with evaluable samples are given above each bar. Pre-existing Ad4 immunity was defined as an Ad4 microneutralisation titre greater than 6 before initial vaccination.

Ad4 immunity at baseline (neutralising antibody >6) was noted in 35 (28%) of 125 of vaccinees and was associated with lower occurrence of vaccine take, pre-boost cellular immune response, and post-boost HAI and microneutralisation antibody response (figure 3). This effect was largely overcome in the high-dose cohorts (10^10^ VP and 10^11^ VP). Similarly, high doses were more likely to outstrip the response from the initial vaccination: recurrence of vaccine virus replication in participants who had had a take with their previous vaccination increased with increasing dose. Recurrence (second take) of Ad4-H5-Vtn replication after second or third vaccination occurred in 32 (36%) of 89 vaccinees who had had a take during a previous vaccination, ranging from 14% (three of 21) with 10^8^ VP to 65% (15 of 23) with 10^11^ VP.

## Discussion

The Ad4-H5-Vtn vector vaccine was well tolerated; symptoms of reactogenicity were generally mild and resolved within 7 days of vaccination. No serious treatment-related adverse events occurred. Ad4-H5-Vtn was not detected by PCR analysis of samples from household contacts of participants. After three vaccinations, haemagglutinin-specific antibody responses to all doses were scarce and of low titres, with HAI seroconversion ranging from 4% to 19% among the vaccine cohorts, compared with 7% among placebo recipients. However, after one inactivated parenteral H5N1 immunisation, HAI seroconversion occurred in 80% of participants (100% in the highest-dose cohort), compared with 36% of placebo recipients, and GMT was significantly higher in participants who had been primed with Ad4-H5-Vtn than in those who had received placebo (panel).

Panel **Research in context**

**Systematic review**

We searched the medical literature (PubMed, literature cited in relevant publications) for clinical studies of replication-competent vector vaccines, avian influenza vaccines, and Ad4 or Ad7 vaccines. The PubMed search used the terms “pandemic influenza”, “replication competent vector”, “adenovirus 4”, and “vector vaccine”; it was not limited by date or language. The date of the last search was Feb 21, 2012. We noted that few replicating vector vaccines have progressed to clinical assessment, and that most, while perhaps priming and inducing a cellular immune response to transgene expressed antigens, did not induce robust antibody responses. We noted that H5N1 vaccines, either inactivated or live attenuated, generally did not induce robust antibody responses compared with influenza vaccines, either seasonal or other avian influenza strains, such as H9N2 or H7N3.

**Interpretation**

As far as we are aware, our study is the first clinical trial of a replication-competent Ad4 vector vaccine for any indication, and one of the few replication-competent vector clinical studies for influenza. We confirmed that the orally administered Ad4 H5N1 vector vaccine seemed to have a safety profile, including low transmissibility, similar to the parent Ad4 vaccine currently used by the US military. Similar to other vector vaccines, the Ad4 H5N1 vector induced a cellular immune response but only minimum antibody response. However, one boost with inactivated H5N1 vaccine led to robust haemagglutination inhibition and H5N1 neutralising antibody responses. This study provides evidence supporting the oral replicating Ad4 vector prime-inactivated boost as a vaccine approach for pandemic influenza, and potentially other infections for which both cellular and antibody responses are needed. However, confirmatory studies will be needed to better assess doses and intervals between prime and boost.

The Ad4-H5-Vtn vector was bioengineered by partial deletion in the E3 region and insertion of the H5 haemagglutinin gene. The function of the E3 region in Ad4 is unknown, but, on the basis of strong E3 homology with Ad5, it is thought to be important in modulation of host-antiviral responses.^22^ Since Ad4 does not replicate in non-human animals except chimpanzees,^23^ replication was assessed in human cell lines before we made an investigational new drug application and was shown to be attenuated compared with unmodified Ad4.^12^ Nevertheless, the recorded occurrence of Ad4 seroconversion and GMTs in both Ad4 seronegative and seropositive Ad4-H5-Vtn recipients with doses of 10^9^ VP–10^11^ VP (appendix) are much the same or higher than in the US military Ad4/Ad7 vaccine phase 1 study.^13^ At these high doses, Ad4-H5-Vtn induced haemagglutinin-specific cellular responses and primed for HAI antibody responses, even in participants with pre-existing Ad4 immunity.

The Ad4-H5-Vtn vaccine also resembled the US military Ad4 vaccine with respect to safety and a low rate of transmissibility.^11^, ^24^, ^25^, ^26^ Although asymptomatic transmission to intimate household contacts and children had been noted in studies before licensure, the US military Ad4 vaccine did not seem to be transmitted among military recruits.^11^, ^24^, ^25^, ^26^ In our study of Ad4-H5-Vtn, no dose limiting or notable toxic effects were identified with doses ranging from 10^7^ VP to 10^11^ VP. All household contacts were enrolled into the study and monitored for potential acquisition of Ad4-H5-Vtn infection. No confirmed transmission to these contacts occurred, although asymptomatic seroconversion to Ad4 was recorded in two contacts of vaccinees and one contact of a placebo recipient. These were presumed seroconversions to wild-type Ad4, since Ad4-H5-Vtn was not detected in throat or rectal swabs of these three household contacts, but seroconversion attributable to Ad4-H5-Vtn infection cannot be ruled out in the household contacts of the two vaccinees. Transmission to contacts of vaccinees is always a potential concern for live vaccines. Although the safety record of the US military Ad4 and Ad7 vaccines, including safety in contacts of vaccinees,^11^, ^24^, ^25^, ^26^ and the absence of confirmed transmission in our study provide reassurance, assessment of transmissibility to, and safety in, vulnerable contacts will require further studies.

The Ad4-H5-Vtn vaccine induced haemagglutinin-specific cellular immune responses but only primed for haemagglutinin-specific antibody responses. However, after one inactivated H5N1 parenteral vaccine boost, we noted substantial HAI and neutralising antibody responses with occurrence of seroconversion and GMTs higher than achieved with unadjuvanted, inactivated H5N1 vaccines.^14^, ^27^ For example in another study,^14^ after two doses of 90 μg inactivated H5N1 subvirion vaccine only 58% of participants achieved the prespecified protective HAI titre of 1/40 or more and 57% seroconverted. By contrast, we showed that after priming with Ad4-H5-Vtn and boosting with inactivated H5N1 vaccine, post-boost seroprotection and seroconversion were 80% and 80% with 10^10^ VP and 89% and 100% with 10^11^ VP. These proportions are higher^28^ or similar^29^, ^30^, ^31^ to seroprotection rates for adjuvanted H5N1 vaccines or DNA priming followed by inactivated H5N1 boosting.^32^ In the DNA priming study,^32^ post-boost antibody responses were much the same, irrespective of whether vaccinees received one or two priming doses. Although the H5N1 boost was given mostly to volunteers who had received all three doses of the oral Ad4-H5-Vtn, the pattern of cellular immune response and vaccine take, both occurring predominantly after the first Ad4-H5-Vtn administration, suggest that one oral dose would have been sufficient for priming. A phase 2 study to confirm that one dose of Ad4-H5-Vtn is sufficient to prime for robust post-boost antibody responses, to investigate dosing of boost vaccine, and to assess the potential for broadening of response by cross-clade boosting, is being planned. Although speculation about the relative advantages and disadvantages of the oral Ad4 replicating vector approach versus DNA or replication-defective vectors might be premature, potential advantages, such as ease and reduced costs both of storage and of self-administration for an oral vaccine formulated as a capsule or tablet might be important in a national or worldwide response to an avian influenza pandemic. Though safety concerns about replicating vectors exist, the safety of the parent Ad4 vaccine virus, well established over many years by the US military,^11^ provides some reassurance.

The profile of immune response to the oral Ad4-H5-Vtn prime—ie, low occurrence of HAI or neutralising antibody responses, but significant H5 haemagglutinin-specific cellular responses—resembles responses to live-attenuated H5N1, H5N2, and H6N1 intranasal candidate vaccines.^33^, ^34^, ^35^ We do not know whether, in the absence of haemagglutinin-protein boosting, the predominantly cellular immune response induced by Ad4-H5-Vtn, or a rapid anamnestic antibody response on exposure to H5N1, would be sufficient to protect against H5N1 infection or disease. The correlates of protection for live-attenuated seasonal influenza vaccine are not well understood, particularly in children, and some reports suggest protection is mediated by local mucosal, cellular immune responses, or both, and that antibody titres much higher than the conventional HAI titre of at least 1/40 are required.^36^, ^37^, ^38^ Thus, we do not know whether one or several doses of the Ad4-H5-Vtn would be protective without parenteral boost, or whether the antibody response engendered by a prime-boost approach would be protective. Nevertheless, on the basis of our results, we might be able to infer that for pandemic H5N1 influenza, one oral Ad4-H5 priming dose, followed by a parenteral boost, would give better antibody response rates than two doses of inactivated vaccine, and, unlike the conventional parenteral vaccines, induce a robust cellular immune response. Potential benefits, such as a reduction of the number of doses of inactivated pandemic influenza vaccine required to be manufactured in a short time, and possibly prepandemic priming, have been suggested for the prime-boost approach.^39^

A possible reason why replicating Ad4-H5-Vtn by itself did not induce a good haemagglutinin-specific antibody response is that the H5 haemagglutinin antigen intrinsically is a poor immunogen, and that an Ad4 vector with a different transgene encoded antigen might have induced robust antibody responses without boost. Experience with other avian influenza vaccines is consistent with poor intrinsic immunogenicity of the H5 haemagglutinin. Two dose regimens of live-attenuated versions of H5N1 or H5N2 induced HAI antibody responses in only 10%^34^ or 20%^33^ of volunteers, whereas analogous live-attenuated avian influenza H9N2 and H7N3 vaccines had responses as high as 92% and 62%, respectively.^40^, ^41^

Cell tropism attributable to inherent or unique properties of the Ad4 vector virus remains another possible explanation. However, in chimpanzees in which Ad4 vectors replicate to a limited extent, they have induced good HIV-Env antibody responses,^42^ and in other animals, including non-human primates, in which Ad4 vectors perform as a replication-defective vector, robust HIV-Env antibody or HAI responses have also been induced.^12^, ^43^ Another possible explanation is that replicating vectors might inherently express their transgenes in a manner not conducive to direct stimulation of antibody. Analogous results (ie, marginal antibody response to the transgene encoded antigen but significant priming for subsequent boost) were noted with a replication-competent vaccinia vector expressing HIV gp160,^7^, ^8^ but safety issues with vaccinia have precluded its wide development.^44^ Other replicating viral vectors have induced cellular responses, but only poor antibody responses in clinical trials.^6^

Several potential explanations exist for the low haemagglutinin-specific binding antibody and sporadic HAI responses, despite haemagglutinin-specific cellular responses. Oral administration of the Ad4-H5-Vtn vector efficiently primed for HAI antibody responses as evidenced by the robust antibody responses to boosting with inactivated parenteral H5N1 vaccine. Since heamagglutinin-specific cellular responses were induced, which would presumably provide helper functions, and low level haemagglutinin-specific binding antibody was also generated, it is likely that sufficient antigen might not have been available for robust B-cell activation and maturation. Insufficient haemagglutinin antigen presentation might have several causes including insufficient amounts of expression, suboptimum conformation (which would have a lesser effect on T-cell responses), lack of transport of expressed antigen to locations available for sampling by antigen-presenting cells and B cells, or degradation of the antigen. Further human experience with these Ad4 recombinant vectors expressing either *Bacillus anthracis* protective antigen, HIV-1 mosiac gag, or HIV-1 envelope will come from phase 1 trials in 2013, and the results might show whether immune profiles noted in this trial were the result of the characteristics of the H5 antigen or inherent properties of the oral administration of these replication-competent vectors.

Irrespective of the cause for the poor antibody response after oral administration of the Ad4-H5-Vtn vaccine, its immunogenicity—vector induction of a cellular immune response but with antibody response only after heterologous protein boosting—is the profile that was noted after immunisation with a canarypox vector and rEnv boost in the only HIV vaccine trial to have shown any, if only modest, efficacy.^45^ A vaccination regimen consisting of priming by a replicating Ad4 HIV-Env vector, followed by rEnv parenteral boosting might provide a similar or better immunological profile, particularly if oral priming by adenovirus leads to a mucosal cellular response as shown in animals.^1^, ^46^, ^47^ Replication-competent vector vaccines have, for many years, been proposed as potentially being the best avenue to a successful HIV-1 vaccine, since they are deemed to approximate more closely the features of live-attenuated vaccines, induce HIV-specific immune responses at the virus mucosal entry point, and might have a stronger immunogenicity than non-replicating vectors by induction of more potent innate and adaptive immune responses.^2^ Ad4 vaccine vectors expressing HIV-1 Env and Gag, to be followed by rEnv boost are currently scheduled for phase 1 clinical trials in 2013. Replicating Ad4 vector vaccines might also be effective in prime-boost regimens for other infections such as malaria, herpes simplex virus, and cytomegalovirus, for which both cellular and antibody responses seem to be needed for protection.

## References

[1] Robert-Guroff M (2007). Replicating and non-replicating viral vectors for vaccine development. Curr Opin Biotechnol.

[2] Excler JL, Parks CL, Ackland J, Rees H, Gust ID, Koff WC (2010). Replicating viral vectors as HIV vaccines: summary report from the IAVI-sponsored satellite symposium at the AIDS vaccine 2009 conference. Biologicals.

[3] Falzarano D, Geisbert TW, Feldmann H (2011). Progress in filovirus vaccine development: evaluating the potential for clinical use. Expert Rev Vaccines.

[4] Kopecky-Bromberg SA, Palese P (2009). Recombinant vectors as influenza vaccines. Curr Top Microbiol Immunol.

[5] Barouch DH (2010). Novel adenovirus vector-based vaccines for HIV-1. Curr Opin HIV AIDS.

[6] Bernstein DI, Malkin E, Abughali N (2012). Phase 1 study of the safety and immunogenicity of a live, attenuated respiratory syncytial virus and parainfluenza virus type 3 vaccine in seronegative children. Pediatr Infect Dis J.

[7] Cooney EL, Collier AC, Greenberg PD (1991). Safety of and immunological response to a recombinant vaccinia virus vaccine expressing HIV envelope glycoprotein. Lancet.

[8] Cooney EL, McElrath MJ, Corey L (1993). Enhanced immunity to human immunodeficiency virus (HIV) envelope elicited by a combined vaccine regimen consisting of priming with a vaccinia recombinant expressing HIV envelope and boosting with gp160 protein. Proc Natl Acad Sci USA.

[9] Profectus BioSciences I (2012). First human volunteer immunized in clinical trial of Profectus BioSciences' vesicular stomatitis virus-vectored HIV-1 vaccine. http://www.profectusbiosciences.com/news_press.html.

[10] Tacket CO, Losonsky G, Lubeck MD (1992). Initial safety and immunogenicity studies of an oral recombinant adenohepatitis B vaccine. Vaccine.

[11] Gaydos CA, Gaydos JC (1995). Adenovirus vaccines in the US military. Mil Med.

[12] Alexander J, Ward S, Mendy J (2012). Pre-clinical evaluation of a replication-competent recombinant adenovirus serotype 4 vaccine expressing influenza H5 hemagglutinin. PLoS One.

[13] Lyons A, Longfield J, Kuschner R (2008). A double-blind, placebo-controlled study of the safety and immunogenicity of live, oral type 4 and type 7 adenovirus vaccines in adults. Vaccine.

[14] Treanor JJ, Campbell JD, Zangwill KM, Rowe T, Wolff M (2006). Safety and immunogenicity of an inactivated subvirion influenza A (H5N1) vaccine. N Engl J Med.

[15] Boyce TG, Hsu HH, Sannella EC (2000). Safety and immunogenicity of adjuvanted and unadjuvanted subunit influenza vaccines administered intranasally to healthy adults. Vaccine.

[16] Kozlowski PA, Lynch RM, Patterson RR, Cu-Uvin S, Flanigan TP, Neutra MR (2000). Modified wick method using Weck-Cel sponges for collection of human rectal secretions and analysis of mucosal HIV antibody. J Acquir Immune Defic Syndr.

[17] WHO (2002). Manual on animal influenza diagnosis and surveillance.

[18] McCullers JA, Van De Velde LA, Allison KJ, Branum KC, Webby RJ, Flynn PM (2010). Recipients of vaccine against the 1976 “swine flu” have enhanced neutralization responses to the 2009 novel H1N1 influenza virus. Clin Infect Dis.

[19] Crawford-Miksza LK, Schnurr DP (1994). Quantitative colorimetric microneutralization assay for characterization of adenoviruses. J Clin Microbiol.

[20] Rowe T, Abernathy RA, Hu-Primmer J (1999). Detection of antibody to avian influenza A (H5N1) virus in human serum by using a combination of serologic assays. J Clin Microbiol.

[21] Lewis JJ, Janetzki S, Schaed S (2000). Evaluation of CD8(+) T-cell frequencies by the Elispot assay in healthy individuals and in patients with metastatic melanoma immunized with tyrosinase peptide. Int J Cancer.

[22] Ilan Y, Droguett G, Chowdhury NR (1997). Insertion of the adenoviral E3 region into a recombinant viral vector prevents antiviral humoral and cellular immune responses and permits long-term gene expression. Proc Natl Acad Sci USA.

[23] Chengalvala M, Lubeck MD, Davis AR (1991). Evaluation of adenovirus type 4 and type 7 recombinant hepatitis B vaccines in dogs. Vaccine.

[24] Stanley ED, Jackson GG (1969). Spread of enteric live adenovirus type 4 vaccine in married couples. J Infect Dis.

[25] Lichtenstein DL, Wold WS (2004). Experimental infections of humans with wild-type adenoviruses and with replication-competent adenovirus vectors: replication, safety, and transmission. Cancer Gene Ther.

[26] Mueller RE, Muldoon RL, Jackson GG (1969). Communicability of enteric live adenovirus type 4 vaccine in families. J Infect Dis.

[27] Ehrlich HJ, Muller M, Oh HM (2008). A clinical trial of a whole-virus H5N1 vaccine derived from cell culture. N Engl J Med.

[28] Lopez P, Caicedo Y, Sierra A, Tilman S, Banzhoff A, Clemens R (2011). Combined, concurrent, and sequential administration of seasonal influenza and MF59-adjuvanted A/H5N1 vaccines: a phase II randomized, controlled trial of immunogenicity and safety in healthy adults. J Infect Dis.

[29] Leroux-Roels I, Borkowski A, Vanwolleghem T (2007). Antigen sparing and cross-reactive immunity with an adjuvanted rH5N1 prototype pandemic influenza vaccine: a randomised controlled trial. Lancet.

[30] Lasko B, Reich D, Madan A, Roman F, Li P, Vaughn D (2011). Rapid immunization against H5N1: a randomized trial evaluating homologous and cross-reactive immune responses to AS03(A)-adjuvanted vaccination in adults. J Infect Dis.

[31] Levie K, Leroux-Roels I, Hoppenbrouwers K (2008). An adjuvanted, low-dose, pandemic influenza A (H5N1) vaccine candidate is safe, immunogenic, and induces cross-reactive immune responses in healthy adults. J Infect Dis.

[32] Ledgerwood JE, Wei CJ, Hu Z (2011). DNA priming and influenza vaccine immunogenicity: two phase 1 open label randomised clinical trials. Lancet Infect Dis.

[33] Chirkova TV, Naykhin AN, Petukhova GD (2011). Memory T-cell immune response in healthy young adults vaccinated with live attenuated influenza A (H5N2) vaccine. Clin Vaccine Immunol.

[34] Karron RA, Talaat K, Luke C (2009). Evaluation of two live attenuated cold-adapted H5N1 influenza virus vaccines in healthy adults. Vaccine.

[35] Talaat KR, Karron RA, Luke CJ (2011). An open label phase I trial of a live attenuated H6N1 influenza virus vaccine in healthy adults. Vaccine.

[36] Black S, Nicolay U, Vesikari T (2011). Hemagglutination inhibition antibody titers as a correlate of protection for inactivated influenza vaccines in children. Pediatr Infect Dis J.

[37] Forrest BD, Pride MW, Dunning AJ (2008). Correlation of cellular immune responses with protection against culture-confirmed influenza virus in young children. Clin Vacc Immunol.

[38] Osterholm MT, Kelley NS, Sommer A, Belongia EA (2012). Efficacy and effectiveness of influenza vaccines: a systematic review and meta-analysis. Lancet Infect Dis.

[39] Lu S (2011). Two is better than one. Lancet Infect Dis.

[40] Karron RA, Callahan K, Luke C (2009). A live attenuated H9N2 influenza vaccine is well tolerated and immunogenic in healthy adults. J Infect Dis.

[41] Talaat KR, Karron RA, Callahan KA (2009). A live attenuated H7N3 influenza virus vaccine is well tolerated and immunogenic in a Phase I trial in healthy adults. Vaccine.

[42] Lubeck MD, Natuk RJ, Chengalvala M (1994). Immunogenicity of recombinant adenovirus-human immunodeficiency virus vaccines in chimpanzees following intranasal administration. AIDS Res Hum Retroviruses.

[43] Gomez-Roman VR, Robert-Guroff M (2003). Adenoviruses as vectors for HIV vaccines. AIDS Rev.

[44] Letvin NL (2005). Progress toward an HIV vaccine. Annu Rev Med.

[45] Rerks-Ngarm S, Pitisuttithum P, Nitayaphan S (2009). Vaccination with ALVAC and AIDSVAX to prevent HIV-1 infection in Thailand. N Engl J Med.

[46] Ko SY, Cheng C, Kong WP (2009). Enhanced induction of intestinal cellular immunity by oral priming with enteric adenovirus 41 vectors. J Virol.

[47] Wang L, Cheng C, Ko SY (2009). Delivery of human immunodeficiency virus vaccine vectors to the intestine induces enhanced mucosal cellular immunity. J Virol.

