# Supplementary material for: Safety and immunogenicity of an oral, replicating adenovirus serotype 4 vector vaccine for H5N1 influenza: a randomised, double-blind, placebo-controlled, phase 1 study
Source: Lancet Infect Dis. 2013 Mar;13(3):238–59. doi: 10.1016/S1473-3099(12)70345-6 (PMC3576519; doi:10.1016/S1473-3099(12)70345-6)
Supplement: Supplementary appendix [file mmc1.pdf]

THE LANCET Infectious Diseases

Supplementary webappendix

This webappendix formed part of the original submission and has been peer reviewed.
We post it as supplied by the authors.

Supplement to: Gurwith M, Lock M, Taylor EM, et al. Safety and immunogenicity of an oral, replicating adenovirus serotype 4 vector vaccine for H5N1 influenza: a randomised, double-blind, placebo-controlled, phase 1 study. *Lancet Infect Dis* 2013; published online Jan 29. http://dx.doi.org/10.1016/S1473-3099(12)70345-6

Supplementary appendix

Study sites

The Center for Pharmaceutical Research, 1010 Carondelet Drive, Suite 426, Kansas City, MO 64114.
University of Kentucky School of Medicine, Department of Medicine, Room MN-672, 800 Rose Street, Lexington, KY 40536-0084.
Coastal Carolina Research, Inc., 1156 Bowman Rd, Suite 102; Mt. Pleasant, SC 29464.
University of Rochester School of Medicine and Dentistry, 601 Elmwood Ave, Rochester, New York 14642.

Methods

Vaccine The vaccine vector virus, designated as Ad4-H5-Vtn, is a recombinant, replication-competent Ad4, encoding full-length hemagglutinin (HA) from H5N1 influenza virus (A/Vietnam/1194/2004)[1]. The recombinant virus was derived from the US Military Ad4 vaccine virus which had been originally isolated from a military recruit with acute respiratory disease before being passaged in WI-38 cells[2]. The H5 HA transgene was modified to include a specific deletion/mutation of the polybasic protein cleavage domain (ΔPCS) that blocks cleavage of full-length HA into the active subunits[3]. This mutation is required in the generation of traditional, inactivated H5N1 virus vaccines. The H5 gene was cloned into a minor deletion in the E3 region of Ad4 which increased the cloning capacity while preserving the known functions of E3.The expression cassette was modified to include sequences that would link the expression of H5-HA with the adenovirus major late promoter, which exerts its activity over a long range by carefully controlled RNA splicing.

Recombinant virus sequences were generated in a bacterial plasmid containing the complete Ad4 genome and which was used to transfect MRC-5 from a master cell bank. Once virus was identified by CPE, the lysate was expanded and plaque purified twice before entering GMP manufacturing. Following anion exchange chromatography and tangential flow filtration, the purified virus was lyophilized and formulation as enteric-coated capsules for oral delivery. The capsules are stable when stored at -20 °C for extended periods. The purified virus concentration was determined by analytical anion-exchange HPLC[4], with the viral particle number (vp) being used to define the final capsule dosage. The dosage range explored in this Phase 1 study was 10^7 to 10^{11} vp, and the vaccine was administered as either a single capsule (10^7 to 10^{10} dosage cohorts) or 10 capsules (10^{11} dosage cohort). Placebo capsules were filled with sucrose, the pre-dominant constituent of the lyophilized vaccine, and were stored under the same conditions. For reference, the US Military Ad4 vaccine is currently dosed as "no less than" 4·5 $\log_{10}$ $TCID_{50}$, which we estimate to be a dosage greater than 10^7 vp[2].

Study design

This was an ascending dosage, double-blind placebo-controlled study in which 5 cohorts of at least 32 subjects were enrolled. Subjects were recruited into each cohort in a dose-escalating manner and were randomly assigned to vaccine or placebo (vaccine:placebo=3:1) within each cohort. Subjects in each cohort received up to 3 doses of Ad4-H5-Vtn (10^7, $10^{8,}$ 10^9, 10^{10}, 10^{11} vp/dose) or placebo, each dose given approximately 56 days apart. Enrollment was restricted to healthy men and non-pregnant women, ages 18 to 40. Based on experience with the military Ad4 vaccine, intestinal replication and shedding of the Ad4-H5-Vtn virus and possible transmission of the vaccine virus was anticipated and therefore enrollment was restricted to subjects who either lived alone or had no more than two healthy, adult (ages 18-65) household contacts (HHCs) who were also willing to be enrolled in the study in order to evaluate potential transmission of Ad4-H5-Vtn virus.

Escalation of dosage and administration of a second or third dose within a cohort was determined by a systematic and independent safety review. When clinical and virological data through 14 days post-vaccination was available from at least 90% of the Ad4-H5-Vtn recipients and associated HHCs, the data was reviewed by PaxVax and an independent safety, data monitoring committee (DMC) with a focus on the occurrence of any severe adverse events, evidence of systemic spread of the Ad4-H5-Vtn vaccine virus, or evidence of potential Ad4-related disease in the

HHCs. If there were no significant safety issues identified, the subjects received their second or third dose administration, and the next, higher dosage cohort was enrolled.

Safety in vaccinated subjects was monitored by documenting solicited reactogenicity using a 7-day subject diary after each vaccination and unsolicited adverse events throughout the study. In addition, clinical laboratory tests (hematology, chemistry, and urinalysis) were conducted at baseline and 7-days after each vaccination. Rectal swabs, throat swabs, and blood were collected for evaluation of Ad4-H5-Vtn virus shedding by Ad4-H5-Vtn-specific PCR; any samples that were positive for Ad4-H5-Vtn by PCR were further evaluated in an Ad4-H5-Vtn-specific culture assay. Safety in HHCs was monitored by documentation of adverse events and by evaluating potential transmission of Ad4-H5-Vtn virus by PCR (rectal and throat swabs) 14 days after each immunization in the vaccinee, and by Ad4 and H5 HA seroconversion with blood specimens at 56 days after each immunization in the vaccinee.

Additional physical examinations, safety laboratory tests, and pregnancy tests were performed on any Vaccinee or HHC, if indicated by the results of scheduled safety assessments or if they presented with signs or symptoms of potential adenovirus syndromes, such as fever, upper respiratory illness, nephritis/cystitis, conjunctivitis, or diarrhea. These tests included surveillance for Ad4-H5-Vtn infection by PCR, and then culture, if the PCR were positive of blood, rectal and throat swabs, and of other appropriate clinical specimens such as urine or conjunctival swabs.

Immunological and virological assays

Hemagglutination-inhibition (HAI): The H5N1 HAI assay was performed by Focus Diagnostics, Irvine, CA using a modification of the WHO method[5]. Prior to the HAI assay, all serum samples from human subjects were treated with receptor destroying enzyme (RDE) overnight at 37°C and determined to be free of non-specific inhibitors. Serial 2-fold dilutions of sera were incubated for 20 minutes at 37°C with the fixed concentration of 4 hemagglutination units (4 HAU) of standard H5N1 antigen (inactivated recombinant H5N1 influenza A virus, source: NIBSC). A 0·75% suspension of horse red blood cells (hRBC, Lampire, Pipersville, PA) was then added to each well and incubated at room temperature for 2 hours. The HAI titer was determined as the reciprocal of the last dilution of sera that completely inhibited hemagglutination. Wells containing hRBC only (no virus or serum), hRBC + 4 HAU virus (no serum), virus backtitration, and testing of positive and negative control sera were used in each assay as quality controls.

Ad4 neutralization: The Ad4 microneutralization assay was performed by Focus Diagnostics. Serial dilutions (1:4 to 1:8192) of the test sera were incubated for approximately one hour with a known concentration of reference virus. A549 cells were then added to the virus serum neutralization mix and incubated for 7 days. The adenovirus neutralizing antibody was then detected and quantitated by the use of a colorimetric assay[6]. The neutralizing titer 50 (NT50) of the test sera is measured by determining the serum dilution that gives a 50% protection in CPE when compared to the cell-only control. Samples were tested in 6 repeats and NT50 was determined by use of the Karber formula.

H5N1 microneutralization (MN)[7]: The MN assay was performed in the laboratory of Richard Webby, St. Jude's, Memphis, TN. Serum levels of H5-specific neutralizing antibody were detected by microneutralization assay on MDCK cells grown to near confluency in 96-well plates. One hundred 50% tissue culture infectious doses of the attenuated A/Vietnam/1203/2004 (H5N1) vaccine virus was incubated with serial two-fold dilutions of each serum sample. Serum and virus mixtures were done in triplicate and incubated at 37°C for 1 hr. After incubation, the growth media was removed from the MDCK cells and the serum-virus mixes added. After a further 1hr incubation at 37°C, the mixes were aspirated from each well of the 96-well plate, the cells washed twice in PBS, then fresh media containing 1mg/ml TPCK-treated trypsin was added to each well. Plates were incubated for 72 hours at 37°C and the presence of virus determined by hemagglutination assay with chicken red blood cells. Neutralizing titers were expressed as the reciprocal of the serum dilution that inhibited 50% of the growth.

HA-specific ELISA[8]: The HA-specific ELISA antibody binding assay was performed at PaxVax. Blood samples were collected from subjects using Vacutainer tubes containing sodium heparin, shipped overnight to PaxVax, and processed for plasma following standard procedures. Plasma was stored at -20 °C until use in the ELISA. Plasma samples were tested for IgG and IgA antibody specific for hemagglutinin (HA) protein from A/Vietnam/1203/2004 influenza (H5N1) virus by ELISA using endpoint titration. EIA/RIA high binding immunoassay 96-well plates (Corning, Lowell, MA) were coated with 100 µL of recombinant H5 HA protein (eEnzyme, Gaithersburg, MD) diluted to 1 µg/mL in PBS and incubated overnight at 4 °C. H5 HA antigen-coated ELISA plates were washed 6 times with wash buffer (PBS, 0·05% tween20), followed by addition of 200 µL 10% normal goat serum (Life Technologies, Calsbad, CA) to each well, and plates incubated for 1 hour at 37 °C. ELISA plates were then washed 6 times with wash buffer and 100 µL of 10% normal goat serum added to each well. Plasma samples were diluted 1/6·25 in 10% normal goat serum containing 0·5% gelatin and 0·15% tween20 and incubated for 1 hour at 37 °C prior to addition to 96-well plates. One hundred microliters of diluted plasma was added to the first row of the ELISA plates (1/12·5 final dilution) and the plasma diluted two-fold from 1/12·5 to 1/12800. For plasma samples obtained following Sanofi vaccine boost immunization, samples were further diluted from 1/12·5 to 1/3276800. After 1·5 hour incubation, plates were washed 6 times with wash buffer and 100 µL of horseradish peroxidase (HRP)-labeled goat anti-human IgG or IgA antibody (Calbiochem, San Diego, CA) diluted 1/10000 in 10% normal goat serum were added to each well. The plates were incubated for 1 hour at room temperature and washed 6 times with wash buffer. One hundred microliters of TMB single solution substrate (Life Technologies) was added to each well and reaction stopped with an equal volume of 1N HCl after 3 minutes. The absorbance was measured at 450 nm with either a BMG Fluostar microplate reader (BMG Labtech, Cary, NC) or Spectromax M3 spectrophotometer microplate reader (Molecular Devices, Sunnyvale, CA) and analyzed using Excel (for endpoint determination) and Graphpad Prism (for EC_{50}) analysis software. The IgG or IgA antibody endpoint titer for test plasma was calculated as the reciprocal of the highest dilution of plasma that gave an absorbance value greater than the mean A_{450} plus 3 standard deviations. Plasma samples were considered to have a positive IgG or IgA antibody response following vaccination if they exhibited all of the following criteria: 1) a 4-fold increase in antibody titer above individual baseline titer, 2) a 4-fold increase above cohort baseline titer geometric mean, and 3) a 4-fold EC_{50} increase above individual baseline EC_{50}. The baseline cutoff was designated as the next dilution above the baseline geometric mean.

ELISPOT Assay: HA- and Ad4-specific ELISPOT assays were performed at PaxVax. Blood samples collected in heparinized tubes (Becton-Dickinson, Franklin Lakes, NJ) were shipped by overnight courier and processed the next day for plasma and PBMC. Plasma supernate collected from centrifuged blood samples were stored at -20 °C. PBMC were isolated by Ficoll-Paque (GE Healthcare, Uppsala, Sweden) gradients and cryopreserved in liquid nitrogen in FBS containing 10% DMSO. T-cell response to vaccine antigens were measured in an IFN-γ and IL-2 ELISPOT assay as described[9]. The capture and detection anti-IFN-γ monoclonal antibodies used were clone 1D1K and the clone 7-B6-1 (Mabtech, Mariemont, OH), respectively. For IL-2 responses, antibody from clone 5334 (R&D Systems, Minneapolis, MN) and BG5 (biotinylated, ThermoFisher Pierce Protein; Rockville, IL) were used. PBMC samples from each subject were typically batch tested in the same experiment, always with a pre-vaccination sample to accurately assess differences in pre- and post-vaccine responses. After thawing and resting overnight in culture, PBMC ($2x10^5$ cells/well) were stimulated in triplicate wells with 4 µg/ml of recombinant H5N1 HA protein (Protein Sciences, Meridien, CT) or with 4 pools of overlapping peptides of the HA transgene sequence. Peptides (133 total), 15 residues in length and overlapping by 11 residues, were synthesized and purified by HPLC (>95% purity) by A & A Laboratory (San Diego, CA). Each pool consisted of 33 or 34 peptides total and was tested at a final concentration of 2·5 µg/ml per peptide. As a positive control, each PBMC sample was also tested against the CEF peptide pool (AnaSpec, Fremont, CA)[10] and PHA-P mitogen (Sigma-Aldrich, St. Louis, MO). After incubation for 16-22 hr at 37 °C, wells were washed with PBS-0·05% Tween-20 (Sigma-Aldrich) to remove cells then incubated with biotin-conjugated capture antibody. Spots were subsequently developed by incubation with avidin-peroxidase complex (Vectastain ABC, Vector Laboratories; Burlingame, CA) followed by tetramethylbenzidine (TruBlue peroxidase

substrate, KPL; Gaithersburg, MD). Spots were enumerated with a computer-assisted ELISPOT reader formatted with image capture and counting software (C.T.L.; Shaker Heights, OH). Vaccine induced responses were scored positive if they exceeded 80 net spots/10^6 cells, after subtracting the no-antigen background, and were >4-fold above the pre-vaccine response.

Ad4-H5-Vtn Polymerase Chain Reaction (PCR): PCR testing was designed at PaxVax, and transferred to Focus Diagnostics who performed the assays. The presence of Ad4-H5-Vtn in clinical specimens (throat swabs, rectal swabs and blood serum samples) was detected by a specific real time PCR method with confirmation of a positive result by both the culture and detection of Ad4-H5-Vtn in A549 cells. PaxVax designed primers and a Taqman probe that are specific to the boundary between Ad4 and the 5' H5-Vtn transgene and supplied them to Focus Diagnostics. The Focus method utilized automated extraction of DNA using the Roche MagNA Pure LC System and real time PCR using Taqman chemistry with the appropriate controls including the use of an internal positive amplification control (IPC) is included with each specimen. Any PCR positive samples were further tested by culture on A549 cells and, following visual detection of cytopathic effects (CPE), were fixed and the presence of H5-Vtn detected in cells using an antibody to the hemagglutinin of A/VietNam/1194/2004. Secondary staining with FITC allows the visual detection of infected cells. In approximately 65% of specimens positive by PCR, the Ad4-H5-Vtn virus was identified in this cell culture method.

Statistical methods Safety and immunogenicity were prospectively specified as co-primary objectives. Safety endpoints included solicited signs and symptoms of reactogenicity reported for 7 days post vaccination on a diary card; unsolicited adverse events; and PCR analyses of rectal, throat and blood samples for evidence of excreted or systemic vaccine virus. Household contacts (HHCs) were monitored at regular intervals for evidence of Ad4-H5-Vtn transmission through PCR analyses of rectal and throat samples, and by serum Ad4 microneutralization assay. Adverse events were also collected for HHCs. Two primary immunogenicity endpoints were pre-specified: seroconversion by H5N1 HAI, defined by a 4-fold rise over the pre-vaccination titer, and HAI geometric mean titer (GMT). Additional endpoints included serum H5N1 microneutralization and H5N1 HA-specific ELISA; cellular immune response measured by ELISPOT; serum Ad4 antibody microneutralization; and vaccine "take" defined as either detection of Ad4-H5-Vtn in a rectal sample or Ad4 seroconversion.

The sample size of 24 vaccine recipients per treatment group was determined by the HAI seroconversion endpoint. Specifically, power to detect a two-fold difference in the seroconversion rate between any two treatment groups was estimated at 76% assuming use of a two-sided Fisher's exact test with p=0·05, and assuming the rate in the better performing group was 80%.

Subjects were enrolled in ascending dose cohorts and randomized in 3:1 ratio to either Ad4-H5-Vtn vaccine or placebo. At each site an independent, unblinded pharmacist dispensed either vaccine or placebo according to a computer-generated randomization list with a block size of 8. Subjects, HHCs, investigators, study site personnel, and medical monitors were blinded to subjects' treatment assignment throughout treatment and follow-up. Unblinded administration of the boost vaccination was offered to both vaccine and placebo recipients. Post-randomization stratification was used for analyses of the impact of baseline Ad4 seropositivity.

Pre-boost reactogenicity rates were calculated from the percentage of subjects who reported a specific sign or symptom at least once following any of the three vaccinations with Ad4-H5-Vtn or placebo. The denominator following the first, second, and third vaccinations was the number of subjects who received the first, second, or third vaccination, respectively. Severity rates were determined by the highest severity reported by a subject for a particular sign or symptom. Confidence intervals for percentages were calculated by the Clopper-Pearson method, and pairwise comparisons between percentages were made using Fisher's exact test. Post-boost reactogenicity rates were calculated from the subset of individuals who received the boost vaccination.

Pre-boost rates for vaccine take, seroconversion by either H5N1 or Ad4 antibodies, and cellular immune response were calculated on cumulative basis where the denominator was the number of subjects with a valid baseline measurement for a particular assay and at least one valid post-vaccination result. The denominator for post-boost rates was the number of subjects who had at least one pre-boost measurement and a valid post-boost result.

Confidence intervals for the geometric mean were estimated by log-transforming the data, constructing intervals based on the t distribution in the log domain, and back-transforming the resulting interval to the original scale. Pairwise comparisons of continuous endpoints were made using either t-tests performed on log-transformed data or Wilcoxon tests.

Analyses were specified in a statistical analysis plan. Analyses and sample size estimation were performed using SAS 9·2, S-Plus 7·0, StatXact 8, and nQuery Advisor 5·0.

Safety

Table S-1. List of All Adverse Events of Grade 3 Severity by Treatment, Dose, Subject, and Potential Relationship to Vaccine

ID	System Organ Class	Preferred Term	Related to Vaccine
		Placebo	
01	GENERAL DISORDERS AND ADMINISTRATION SITE CONDITIONS	FATIGUE	Yes
02	HEPATOBILIARY DISORDERS	HYPERBILIRUBINAEMIA	No
03	INVESTIGATIONS	HAEMOGLOBIN DECREASED	No
04	INVESTIGATIONS	HEPATIC ENZYME INCREASED	No
05	MUSCULOSKELETAL AND CONNECTIVE TISSUE DISORDERS	BACK PAIN	No
	MUSCULOSKELETAL AND CONNECTIVE TISSUE DISORDERS	INTERVERTEBRAL DISC PROTRUSION	No
06	GASTROINTESTINAL DISORDERS	PANCREATITIS	No
		Cohort 1: AD4-H5-Vtn 10^7 vp	
11	GENERAL DISORDERS AND ADMINISTRATION SITE CONDITIONS	INJECTION SITE PAIN	Yes
12	NERVOUS SYSTEM DISORDERS	HEADACHE	No
		Cohort 2: AD4-H5-Vtn 10^8 vp	
21	GASTROINTESTINAL DISORDERS	NAUSEA	Yes
	GENERAL DISORDERS AND ADMINISTRATION SITE CONDITIONS	PYREXIA	Yes
	MUSCULOSKELETAL AND CONNECTIVE TISSUE DISORDERS	MYALGIA	Yes
22	INFECTIONS AND INFESTATIONS	GASTROENTERITIS NOROVIRUS	No
23	GENERAL DISORDERS AND ADMINISTRATION SITE CONDITIONS	INJECTION SITE PAIN	No
24	GENERAL DISORDERS AND ADMINISTRATION SITE CONDITIONS	INJECTION SITE PAIN	No
		Cohort 3: AD4-H5-Vtn 10^9 vp	
31	NERVOUS SYSTEM DISORDERS	HEADACHE	Yes
32	INFECTIONS AND INFESTATIONS	UPPER RESPIRATORY TRACT INFECTION	Yes
33	GENERAL DISORDERS AND ADMINISTRATION SITE CONDITIONS	FATIGUE	No
34	NERVOUS SYSTEM DISORDERS	MIGRAINE	No
35	INVESTIGATIONS	HAEMOGLOBIN DECREASED	No
36	PSYCHIATRIC DISORDERS	HALLUCINATION	No
37	GASTROINTESTINAL DISORDERS	TOOTHACHE	No
		Cohort 4: AD4-H5-Vtn 10^{10} vp	

41	GENERAL DISORDERS AND ADMINISTRATION SITE CONDITIONS	PYREXIA	No
42	INFECTIONS AND INFESTATIONS	GASTROENTERITIS VIRAL	No
	Cohort 5: AD4-H5-Vtn 10^{11} vp		
51	PSYCHIATRIC DISORDERS	ANXIETY	No

Severity and potential relationship to vaccine were assessed by blinded investigators at clinical sites. A grade 3 event was considered significant and something that prevented daily activity. There were no grade 4 or 5 events. Events designated as related to vaccine were considered by an investigator to be at least possibly related. ID is a unique pseudo-identifier assigned to each individual in the table.

Table S-2. Percent (95% CI) of Subjects Reporting Solicited Signs and Symptoms of Reactogenicity and Highest Severity Level Reported following Any of Three Vaccinations with either Ad4-H5-Vtn Vaccine or Placebo

Sign or Symptom	Ad4-H5-Vtn Vaccine Recipients						
	**10^7 vp** (N = 24)	**10^8 vp** (N = 25)	**10^9 vp** (N = 27)	**10^{10} vp** (N = 24)	**10^{11} vp** (N = 25)	**All Doses Combined** (N = 125)	**Placebo** (N = 41)
Abdominal Pain							
Any	17 (5,37)	16 (5,36)	19 (6,38)	21 (7,42)	12 (3,31)	17 (11,25)*	2 (0,13)
Mild	8 (1,27)	12 (3,31)	11 (2,29)	17 (5,37)	8 (1,26)	11 (6,18)	2 (0,13)
Moderate	8 (1,27)	4 (0,20)	7 (1,24)	4 (0,21)	4 (0,20)	6 (2,11)	0 (0,9)
Severe	0 (0,14)	0 (0,14)	0 (0,13)	0 (0,14)	0 (0,14)	0 (0,3)	0 (0,9)
Diarrhea							
Any	17 (5,37)	20 (7,41)	15 (4,34)	25 (10,47)	20 (7,41)	19 (13,27)†	5 (1,17)
Mild	12 (3,32)	20 (7,41)	4 (0,19)	17 (5,37)	16 (5,36)	14 (8,21)	5 (1,17)
Moderate	4 (0,21)	0 (0,14)	11 (2,29)	8 (1,27)	4 (0,20)	6 (2,11)	0 (0,9)
Severe	0 (0,14)	0 (0,14)	0 (0,13)	0 (0,14)	0 (0,14)	0 (0,3)	0 (0,9)
Nausea/Vomiting							
Any	4 (0,21)	12 (3,31)	15 (4,34)	4 (0,21)	24 (9,45)	12 (7,19)	7 (2,20)
Mild	0 (0,14)	12 (3,31)	7 (1,24)	4 (0,21)	20 (7,41)	9 (4,15)	7 (2,20)
Moderate	4 (0,21)	0 (0,14)	7 (1,24)	0 (0,14)	4 (0,20)	3 (1,8)	0 (0,9)
Severe	0 (0,14)	0 (0,14)	0 (0,13)	0 (0,14)	0 (0,14)	0 (0,3)	0 (0,9)
Chills							
Any	4 (0,21)	12 (3,31)	4 (0,19)	8 (1,27)	0 (0,14)	6 (2,11)	0 (0,9)
Mild	4 (0,21)	8 (1,26)	4 (0,19)	4 (0,21)	0 (0,14)	4 (1,9)	0 (0,9)
Moderate	0 (0,14)	4 (0,20)	0 (0,13)	4 (0,21)	0 (0,14)	2 (0,6)	0 (0,9)
Severe	0 (0,14)	0 (0,14)	0 (0,13)	0 (0,14)	0 (0,14)	0 (0,3)	0 (0,9)
Muscle/Body Aches							
Any	8 (1,27)	12 (3,31)	22 (9,42)	21 (7,42)	12 (3,31)	15 (9,23)	7 (2,20)
Mild	4 (0,21)	12 (3,31)	15 (4,34)	12 (3,32)	12 (3,31)	11 (6,18)	5 (1,17)
Moderate	4 (0,21)	0 (0,14)	7 (1,24)	8 (1,27)	0 (0,14)	4 (1,9)	2 (0,13)
Severe	0 (0,14)	0 (0,14)	0 (0,13)	0 (0,14)	0 (0,14)	0 (0,3)	0 (0,9)
Joint Pain							
Any	4 (0,21)	0 (0,14)	7 (1,24)	8 (1,27)	4 (0,20)	5 (2,10)	2 (0,13)
Mild	4 (0,21)	0 (0,14)	4 (0,19)	0 (0,14)	4 (0,20)	2 (0,7)	0 (0,9)
Moderate	0 (0,14)	0 (0,14)	4 (0,19)	8 (1,27)	0 (0,14)	2 (0,7)	2 (0,13)
Severe	0 (0,14)	0 (0,14)	0 (0,13)	0 (0,14)	0 (0,14)	0 (0,3)	0 (0,9)
Tiredness							
Any	33 (16,55)	28 (12,49)	26 (11,46)	29 (13,51)	16 (5,36)	26 (19,35)	17 (7,32)
Mild	12 (3,32)	16 (5,36)	7 (1,24)	12 (3,32)	16 (5,36)	13 (7,20)	5 (1,17)
Moderate	21 (7,42)	12 (3,31)	11 (2,29)	17 (5,37)	0 (0,14)	12 (7,19)	10 (3,23)
Severe	0 (0,14)	0 (0,14)	7 (1,24)	0 (0,14)	0 (0,14)	2 (0,6)	2 (0,13)
Headache							
Any	42 (22,63)	28 (12,49)	44 (25,65)	38 (19,59)	24 (9,45)	35 (27,44)	22 (11,38)
Mild	25 (10,47)	20 (7,41)	22 (9,42)	25 (10,47)	16 (5,36)	22 (15,30)	15 (6,29)
Moderate	12 (3,32)	8 (1,26)	19 (6,38)	12 (3,32)	8 (1,26)	12 (7,19)	7 (2,20)
Severe	4 (0,21)	0 (0,14)	4 (0,19)	0 (0,14)	0 (0,14)	2 (0,6)	0 (0,9)
Nasal Congestion/Runny Nose							
Any	54 (33, 74)	32 (15,54)	44 (25,65)	17 (5,37)	16 (5,36)	33 (25,42)‡	15 (6,29)
Mild	33 (16,55)	16 (5,36)	26 (11,46)	12 (3,32)	16 (5,36)	21 (14,29)	12 (4,26)
Moderate	21 (7,42)	16 (5,36)	15 (4,34)	4 (0,21)	0 (0,14)	11 (6,18)	2 (0,13)
Severe	0 (0,14)	0 (0,14)	4 (0,19)	0 (0,14)	0 (0,14)	1 (0,4)	0 (0,9)
Sore Throat							
Any	29 (13,51)	12 (3,31)	22 (9,42)	29 (13,51)	12 (3,31)	21 (14,29)	12 (4,26)
Mild	25 (10,47)	12 (3,31)	11 (2,29)	21 (7,42)	12 (3,31)	16 (10,24)	10 (3,23)
Moderate	4 (0,21)	0 (0,14)	11 (2,29)	8 (1,27)	0 (0,14)	5 (2,10)	2 (0,13)
Severe	0 (0,14)	0 (0,14)	0 (0,13)	0 (0,14)	0 (0,14)	0 (0,3)	0 (0,9)
Cough							
Any	25 (10,47)	8 (1,26)	19 (6,38)	12 (3,32)	4 (0,20)	14 (8,21)	7 (2,20)
Mild	21 (7,42)	8 (1,26)	11 (2,29)	12 (3,32)	4 (0,20)	11 (6,18)	7 (2,20)
Moderate	4 (0,21)	0 (0,14)	7 (1,24)	0 (0,14)	0 (0,14)	2 (0,7)	0 (0,9)
Severe	0 (0,14)	0 (0,14)	0 (0,13)	0 (0,14)	0 (0,14)	0 (0,3)	0 (0,9)
Shortness of Breath							
Any	4 (0,21)	4 (0,20)	4 (0,19)	0 (0,14)	0 (0,14)	2 (0,7)	2 (0,13)
Mild	0 (0,14)	0 (0,14)	0 (0,13)	0 (0,14)	0 (0,14)	0 (0,3)	0 (0,9)
Moderate	4 (0,21)	4 (0,20)	4 (0,19)	0 (0,14)	0 (0,14)	2 (0,7)	2 (0,13)
Severe	0 (0,14)	0 (0,14)	0 (0,13)	0 (0,14)	0 (0,14)	0 (0,3)	0 (0,9)
Fever							

Any	0 (0,14)	4 (0,20)	0 (0,13)	4 (0,21)	0 (0,14)	2 (0,6)	0 (0,9)
Mild	0 (0,14)	4 (0,20)	0 (0,13)	0 (0,14)	0 (0,14)	1 (0,4)	0 (0,9)
Moderate	0 (0,14)	0 (0,14)	0 (0,13)	4 (0,21)	0 (0,14)	1 (0,4)	0 (0,9)
Severe	0 (0,14)	0 (0,14)	0 (0,13)	0 (0,14)	0 (0,14)	0 (0,3)	0 (0,9)

For each category of reactogenicity, "Any" refers to the percent (95% CI) of subjects who reported that symptom at least once following any of the three vaccinations with Ad4-H5-Vtn or placebo. For "Mild," "Moderate," and "Severe", a subject is counted once at the highest severity level he or she reports.
The group comprised of all Ad4-H5-Vtn recipients reported significantly more abdominal pain (p=0·017)*, diarrhea (p=0·027)†, and incidences of nasal congestion/runny nose (p=0·028)‡ than placebo recipients.

Table S-3. Number (%) of Subjects Reporting Solicited Signs or Symptoms of Reactogenicity on 7-day Diary Card Following Boost Vaccination

Reactogenicity Sign or Symptom	Ad4-H5-Vtn All Cohorts (N=83)	Placebo (N=22)
Abdominal Pain	0	0
Diarrhea	0	0
Nausea/Vomiting	1 (1.2%)	2 (9.1%)
Chills	0	2 (9.1%)*
Muscle/Body Aches	7 (8.4%)	2 (9.1%)
Joint Pain	1 (1.2%)	0
Tiredness	1 (1.2%)	2 (9.1%)
Headache	10 (12.0%)	1 (4.5%)
Nasal Congestion/Runny Nose	0	0
Sore Throat	0	0
Cough	0	0
Shortness of Breath	0	0
Injection Site Pain	17 (20.5%)	0†
Injection Site Tenderness	24 (28.9%)	5 (22.7%)
Injection Site Erythema/Redness	3 (3.6%)	0
Injection Site Induration/Swelling	1 (1.2%)	0
Fever	0	0

A vaccinee is counted once within each category if he/she reports reactogenicity at least once in that category. All subjects who received the boost vaccination completed diary cards; percentages are calculated from the number of subjects in each treatment group.
*p=0.042. Both placebo recipients who reported chills graded the symptoms as mild.
†p=0.020. All 17 Ad4-H5-Vtn recipients who reported injection site pain graded it as mild.

Replication of Ad4-H5-vtn vaccine virus

Table S-4. Number (%) of Subjects who had an Ad4-H5-Vtn-specific PCR Positive Rectal Swab by Visit and Number (%) who had their First Positive Sample at each Visit

Study Day	No. of Vacs Rec'd	Ad4-H5-Vtn Cohort 1: 10^7 vp		Ad4-H5-Vtn Cohort 2: 10^8 vp		Ad4-H5-Vtn Cohort 3: 10^9 vp		Ad4-H5-Vtn Cohort 4: 10^{10} vp		Ad4-H5-Vtn Cohort 5: 10^{11} vp	
		Total Positive at Visit	First Time Positive at Visit	Total Positive at Visit	First Time Positive at Visit	Total Positive at Visit	First Time Positive at Visit	Total Positive at Visit	First Time Positive at Visit	Total Positive at Visit	First Time Positive at Visit
7	1 Vac	3 (14%)	3 (14%)	5 (20%)	5 (20%)	5 (19%)	5 (19%)	13 (54%)	13 (54%)	11 (44%)	11 (44%)
14	1 Vac	4 (17%)	1 (4%)	6 (24%)	3 (12%)	4 (15%)	3 (11%)	2 (8%)	1 (4%)	1 (4%)	1 (4%)
28	1 Vac	0	0	0	0	0	0	0	0	0	0
63	2 Vacs	0	0	3 (12%)	3 (12%)	1 (4%)	1 (4%)	1 (5%)	0	2 (9%)	0
70	2 Vacs	4 (17%)	4 (17%)	0	0	0	0	0	0	0	0
84	2 Vacs	0	0	0	0	0	0	0	0	0	0
119	3 Vacs	1 (6%)	1 (6%)	0	0	0	0	0	0	3 (14%)	1 (5%)
126	3 Vacs	0	0	0	0	0	0	0	0	0	0
140	3 Vacs	0	0	0	0	0	0	0	0	0	0

Percentages are calculated from the number of subjects with evaluable samples at each visit.

Table S-5. Cumulative Number (%) of Subjects who Seroconverted by Ad4 Microneutralization and GMT (95% CI) by Visit

Study Day	Number of Vaccinations Received	Ad4-H5-Vtn Cohort 1: 10^7 vp (N=24)	Ad4-H5-Vtn Cohort 2: 10^8 vp (N=25)	Ad4-H5-Vtn Cohort 3: 10^9 vp (N=27)	Ad4-H5-Vtn Cohort 4: 10^{10} vp (N=23)	Ad4-H5-Vtn Cohort 5: 10^{11} vp (N=24)
Cumulative Number (%) Seroconverted						
Day 28	1 Vac	3 (12.5%)	5 (20.0%)	11 (40.7%)	12 (52.2%)	16 (66.7%)
Day 56	1 Vac	3 (12.5%)	6 (24.0%)	12 (44.4%)	14 (60.9%)	18 (75.0%)
Day 84	2 Vacs	6 (25.0%)	10 (40.0%)	19 (70.4%)	18 (78.3%)	22 (91.7%)
Day 112	2 Vacs	7 (29.2%)	12 (48.0%)	19 (70.4%)	18 (78.3%)	22 (91.7%)
Day 140	3 Vacs	7 (29.2%)	12 (48.0%)	19 (70.4%)	18 (78.3%)	23 (95.8%)
GMT (95% CI)						
Day 28	1 Vac	3 (2,5)	14 (6,32)	19 (8,45)	24 (9,65)	30 (12,77)
Day 56	1 Vac	3 (2,5)	15 (7,34)	16 (8,34)	20 (8,53)	29 (12,68)
Day 84	2 Vacs	5 (3,9)	27 (13,54)	24 (11,53)	32 (15,67)	114 (55,237)
Day 112	2 Vacs	6 (3,12)	25 (13,48)	26 (11,59)	31 (14,72)	81 (38,170)
Day 140	3 Vacs	4 (2,8)	25 (13,47)	19 (8,41)	74 (41,135)	151 (66,346)

Seroconversion requires a 4-fold rise over baseline value. Percentages and GMTs are calculated from subjects (N) who had a baseline result and at least one post-vaccination result.

Table S-6. Cumulative Number (%) of Subjects With Pre-existing Ad4 Immunity who Seroconverted by Ad4 Microneutralization and GMT (95% CI) by Visit

Study Day	Number of Vaccinations Received	Ad4-H5-Vtn Cohort 1: 10^7 vp (N=3)	Ad4-H5-Vtn Cohort 2: 10^8 vp (N=10)	Ad4-H5-Vtn Cohort 3: 10^9 vp (N=11)	Ad4-H5-Vtn Cohort 4: 10^{10} vp (N=7)	Ad4-H5-Vtn Cohort 5: 10^{11} vp (N=4)
Cumulative Number (%) Seroconverted						
Day 28	1 Vac	0	1 (10%)	3 (27%)	5 (71%)	1 (25%)
Day 56	1 Vac	0	1 (10%)	4 (36%)	5 (71%)	3 (75%)
Day 84	2 Vacs	0	1 (10%)	7 (64%)	5 (71%)	3 (75%)
Day 112	2 Vacs	0	1 (10%)	7 (64%)	5 (71%)	3 (75%)
Day 140	3 Vacs	0	1 (10%)	7 (64%)	5 (71%)	3 (75%)
GMT (95% CI)						
Day 28	1 Vac	30 (1,1494)	74 (34,161)	74 (15,357)	420 (152,1163)	469 (5,48814)
Day 56	1 Vac	25 (0,2182)	85 (38,193)	79 (36,175)	252 (88,722)	645 (208,2003)
Day 84	2 Vacs	25 (0,3683)	95 (46,195)	107 (46,249)	224 (89,562)	542 (243,1209)
Day 112	2 Vacs	33 (1,1852)	98 (44,218)	100 (40,249)	261 (101,677)	431 (124,1491)
Day 140	3 Vacs	35 (1,961)	79 (41,153)	63 (24,164)	239 (74,775)	527 (333,835)

Pre-existing Ad4 immunity is defined as a positive result (Ad4 titer > 6) at baseline prior to first vaccination. Seroconversion requires a 4-fold rise over baseline value. Percentages and GMTs are calculated from subjects (N) who had a baseline result and at least one post-vaccination result.

Table S-7. Cumulative Number (%) of Subjects Without Pre-existing Ad4 Immunity who Seroconverted by Ad4 Microneutralization and GMT (95% CI) by Visit

Study Day	Number of Vaccinations Received	Ad4-H5-Vtn Cohort 1: 10^7 vp (N=21)	Ad4-H5-Vtn Cohort 2: 10^8 vp (N=15)	Ad4-H5-Vtn Cohort 3: 10^9 vp (N=16)	Ad4-H5-Vtn Cohort 4: 10^{10} vp (N=16)	Ad4-H5-Vtn Cohort 5: 10^{11} vp (N=20)
Cumulative Number (%) Seroconverted						
Day 28	1 Vac	3 (14%)	4 (27%)	8 (50%)	7 (44%)	15 (75%)
Day 56	1 Vac	3 (14%)	5 (33%)	8 (50%)	9 (56%)	15 (75%)
Day 84	2 Vacs	6 (29%)	9 (60%)	12 (75%)	13 (81%)	19 (95%)
Day 112	2 Vacs	7 (33%)	11 (73%)	12 (75%)	13 (81%)	19 (95%)
Day 140	3 Vacs	7 (33%)	11 (73%)	12 (75%)	13 (81%)	20 (100%)
GMT (95% CI)						
Day 28	1 Vac	2 (2,3)	5 (2,11)	7 (3,16)	7 (3,14)	18 (8,38)
Day 56	1 Vac	2 (1,3)	5 (2,11)	5 (2,12)	6 (3,13)	15 (7,32)
Day 84	2 Vacs	4 (2,7)	12 (5,27)	8 (3,20)	15 (8,28)	84 (37,188)
Day 112	2 Vacs	4 (2,7)	12 (5,27)	8 (3,20)	15 (8,28)	84 (37,188)
Day 140	3 Vacs	2 (2,4)	11 (5,23)	8 (3,21)	47 (26,86)	113 (42,300)

Pre-existing Ad4 immunity is defined as a positive result (Ad4 titer > 6) at baseline prior to first vaccination. Seroconversion requires a 4-fold rise over baseline value. Percentages and GMTs are calculated from subjects (N) who had a baseline result and at least one post-vaccination result.

H5 HA-specific IL-2 cellular immune response

Table S-8. Cumulative Percentage (95% CI) of Subjects who had a Positive IL-2 ELISPOT Response and Median SFC (IQR) by Visit

Study Day	No. of Vacs Received	Ad4-H5-Vtn Cohort 1: 10^7 vp	Ad4-H5-Vtn Cohort 2: 10^8 vp	Ad4-H5-Vtn Cohort 3: 10^9 vp	Ad4-H5-Vtn Cohort 4: 10^{10} vp	Ad4-H5-Vtn Cohort 5: 10^{11} vp	Ad4-H5-Vtn All Cohorts Combined	Placebo
Cumulative Percent Positive (95% CI)								
Day 28	1 Vac	13 (3,32)	20 (7,41)	33 (17,54)	39 (20,61)	52 (31,73)	31 (23,40)	2 (0,13)
Day 84	2 Vacs	25 (10,47)	28 (12,49)	41 (22,61)	43 (23,66)	57 (34,77)	39 (30,48)	2 (0,13)
Day 140	3 Vacs	25 (10,47)	36 (18,57)	48 (29,68)	52 (31,73)	57 (34,77)	43 (34,53)	5 (1,17)
Post-boost		23 (5,54)	12 (1,36)	11 (1,35)	33 (12,62)	40 (16,68)	23 (14,34)	33 (15,57)
Median SFC per 10^6 cells (IQR)								
Day 0	0 Vacs	59 (33,115)	72 (51,101)	75 (52,109)	105 (77,179)	68 (49,101)	77 (51,127)	119 (89,154)
Day 28	1 Vac	116 (47,171)	109 (79,216)	148 (108,223)	291 (225,443)	345 (248,527)	189 (108,292)	111 (85,159)
Day 84	2 Vacs	109 (65,180)	121 (97,244)	177 (117,243)	317 (171,407)	268 (203,380)	191 (111,317)	109 (72,178)
Day 140	3 Vacs	129 (45,184)	125 (81,199)	171 (123,239)	244 (175,357)	169 (131,271)	171 (109,259)	103 (59,155)
Post-boost		129 (121,185)	116 (77,209)	107 (59,165)	188 (132,229)	308 (159,620)	111 (75,159)	143 (81,257)

A pre-boost positive response is defined as ≥ 80 spot forming cells (sfc) and 4X greater than baseline sfc, following 1, 2, or 3 vaccinations. A post-boost positive response required ≥ 80 spot forming cells (sfc) and 4X greater sfc than the last post-boost measurement.

H5 HA specific-antibody response

Table S-9. Cumulative Percentage (95% CI) of Subjects who had a Plasma IgG H5 HA-specific ELISA Response and Median Fold-Increase (IQR) by Visit

Study Day	No. of Vacs Received	Ad4-H5-Vtn Cohort 1: 10^7 vp	Ad4-H5-Vtn Cohort 2: 10^8 vp	Ad4-H5-Vtn Cohort 3: 10^9 vp	Ad4-H5-Vtn Cohort 4: 10^{10} vp	Ad4-H5-Vtn Cohort 5: 10^{11} vp	Ad4-H5-Vtn All Cohorts Combined	Placebo
Cumulative Percent Seroconverted (95% CI)								
Pre-boost Vaccinations		N=24	N=25	N=27	N=23	N=24	N=123	N=39
Day 28	1 Vac	8 (1,27)	12 (3,31)	7 (1,24)	22 (7,44)	38 (19,59)	17 (11,25)†	0 (0, 9)
Day 84	2 Vacs	13 (3,32)	12 (3,31)	11 (2,29)	22 (7,44)	46 (26,67)	20 (14,29)†	3 (0, 13)
Day 140	3 Vacs	17 (5,37)	12 (3,31)	11 (2,29)	22 (7,44)	50 (29,71)	22 (15,30)*	5 (1, 17)
Post-boost Vaccination		N=13	N=18	N=18	N=15	N=18	N=82	N=22
Post-boost		69 (39, 91)	72 (47,90)	61 (36,83)	93 (68,100)	100 (81,100)	79 (69,87)*	50 (28,72)
Median Fold-Increase (IQR)								
Day 28	1 Vac	2 (1,6)	2 (1,4)	2 (1,4)	2 (1,4)	4 (2,8)	2 (1,4)§	1 (1, 2)
Day 84	2 Vacs	2 (1,4)	1 (1,2)	1 (1,2)	2 (1,4)	4 (2,8)	2 (1,4)§	1 (1, 1)
Day 140	3 Vacs	4 (2,8)	1 (1,2)	1 (1,2)	2 (1,4)	4 (2,8)	2 (1,4)†	1 (1, 2)
Post-boost		16 (8, 64)	16 (8,32)	4 (4,32)	16 (8,38)	64 (32,128)	16 (8,64)†	8 (4,16)

A pre-boost positive response requires a 4-fold rise over baseline value; post-boost positivity requires a 4-fold rise over the last pre-boost value. Pre-boost fold-increase is the ratio of the value at each pre-boost visit to baseline; post-boost fold increase is the ratio of the post-boost result to the last pre-boost result. IQR is the interquartile range. Pre-boost percentages and fold-increase are calculated from subjects (N) who had a baseline result and at least one post-vaccination result. Post-boost percentages and fold-increases are calculated from subjects (N) who had both pre- and post-boost results. The combined vaccine group was significantly superior to the placebo group at the following levels: $*0.01 \le p < 0.05$; $†0.001 \le p < 0.01$; $§p < 0.001$.

Table S-10. Post-boost Percentage (95% CI) of Subjects who had a Nasal IgG H5 HA-specific kELISA Response and Median Fold-Increase (IQR) over Last Pre-Boost Result

Study Day	Ad4-H5-Vtn Cohort 1: 10^7 vp (N=13)	Ad4-H5-Vtn Cohort 2: 10^8 vp (N=18)	Ad4-H5-Vtn Cohort 3: 10^9 vp (N=18)	Ad4-H5-Vtn Cohort 4: 10^{10} vp (N=14)	Ad4-H5-Vtn Cohort 5: 10^{11} vp (N=19)	Ad4-H5-Vtn All Cohorts Combined (N=82)	Placebo (N=21)
Percent Positive (95% CI)							
Post-boost	85 (55,98)	89 (65,99)	56 (31,78)	93 (66,100)	79 (54,94)	79 (69,87)	62 (38,82)
Median Fold-Increase (IQR)							
Post-boost	3.3 (2.3,5.3)	5.4 (4.3,8.6)	2.4 (1.5,5.4)	6.4 (4.4,9.7)	7.3 (3.6,13.6)	5.1 (2.3,8.6)*	2.7 (1.3,5.1)

A post-boost positive response requires a 2-fold increase over the last pre-boost value. Post-boost fold increase is the ratio of the post-boost result to the last pre-boost result. IQR is the interquartile range. Percentages and GMTs are calculated from subjects (N) who had a pre-boost and post-boost result. The combined vaccine group was significantly superior to the placebo group at the following level: $*p = 0.020$.

Table S-11. Cumulative Percentage (95% CI) of Subjects who had a Plasma IgA H5 HA-specific ELISA Response and Median Fold-Increase (IQR) by Visit

Study Day	No. of Vacs Received	Ad4-H5-Vtn Cohort 1: 10^7 vp	Ad4-H5-Vtn Cohort 2: 10^8 vp	Ad4-H5-Vtn Cohort 3: 10^9 vp	Ad4-H5-Vtn Cohort 4: 10^{10} vp	Ad4-H5-Vtn Cohort 5: 10^{11} vp	Ad4-H5-Vtn All Cohorts Combined	Placebo
Cumulative Percent Seroconverted (95% CI)								
Pre-boost Vaccinations		N=24	N=25	N=27	N=23	N=24	N=123	N=39
Day 28	1 Vac	4 (0,21)	12 (3,31)	7 (1,24)	9 (1,28)	4 (0,21)	7 (3,13)	0 (0,9)
Day 84	2 Vacs	8 (1,27)	20 (7,41)	7 (1,24)	9 (1,28)	13 (3,32)	11 (6,18)	3 (0,13)
Day 140	3 Vacs	17 (5,37)	20 (7,41)	7 (1,24)	9 (1,28)	21 (7,42)	15 (9,22)	5 (1,17)
Post-boost Vaccination		N=13	N=18	N=18	N=15	N=18	N=82	N=22
Post-boost			46 (19-75)	61 (36-83)	50 (26-74)	93 (68-100)	83 (59-96)*	67 (56-77)
Median Fold-Increase (IQR)								
Day 28	1 Vac	1 (1,3)	1 (1,2)	1 (1,2)	2 (1,2)	1 (1,3)	1 (1,2)§	1 (1, 1)
Day 84	2 Vacs	1 (1,2)	1 (1,4)	1 (1,1)	2 (1,2)	2 (1,4)	1 (1,2)	1 (1, 1)
Day 140	3 Vacs	1 (1,2)	2 (1,4)	1 (1,2)	2 (1,2)	2 (1,4)	1 (1,2)*	1 (1, 1)
Post-boost			8 (2,8)	8 (4,16)	8 (2,32)	64 (8,128)	24 (16,32)*	16 (4,32)

A pre-boost positive response requires a 4-fold rise over baseline value; post-boost positivity requires a 4-fold rise over the last pre-boost value. Pre-boost fold-increase is the ratio of the value at each pre-boost visit to baseline; post-boost fold increase is the ratio of the post-boost result to the last pre-boost result. IQR is the interquartile range. Pre-boost percentages and fold-increase are calculated from subjects (N) who had a baseline result and at least one post-vaccination result. Post-boost percentages and fold-increases are calculated from subjects (N) who had both pre- and post-boost results. The combined vaccine group was significantly superior to the placebo group at the following levels: *$0.01 \le p < 0.05$; §$p < 0.001$.

Table S-12. Post-boost Percentage (95% CI) of Subjects who had a Nasal IgA H5 HA-specific kELISA Response and Median Fold-Increase (IQR) over Last Pre-Boost Result

Study Day	Ad4-H5-Vtn Cohort 1: 10^7 vp (N=13)	Ad4-H5-Vtn Cohort 2: 10^8 vp (N=17)	Ad4-H5-Vtn Cohort 3: 10^9 vp (N=18)	Ad4-H5-Vtn Cohort 4: 10^{10} vp (N=14)	Ad4-H5-Vtn Cohort 5: 10^{11} vp (N=19)	Ad4-H5-Vtn All Cohorts Combined (N=81)	Placebo (N=21)
Percent Positive (95% CI)							
Post-boost	31 (9,61)	35 (14,62)	39 (17,64)	57 (29,82)	63 (38,84)	46 (35,57)	29 (11,52)
Median Fold-Increase (IQR)							
Post-boost	1.3 (0.8,2.1)	1.0 (0.3,2.6)	1.5 (0.8,2.8)	2.3 (1.0,7.3)	2.1 (1.4,6.0)	1.7 (0.8,3.2)	1.4 (1.0,2.1)

A post-boost positive response requires a 2-fold increase over the last pre-boost value. Post-boost fold increase is the ratio of the post-boost result to the last pre-boost result. IQR is the interquartile range. Percentages and GMTs are calculated from subjects (N) who had a pre-boost and post-boost result.

Effect of pre-existing Ad4 immunity on post-boost HAI seroconversion

Table S-13. Number (%) of Subjects With Pre-existing Ad4 Immunity who Seroconverted by HAI Post-boost and GMT (95% CI)

Study Day	Ad4-H5-Vtn Cohort 1: 10^7 vp (N=3)	Ad4-H5-Vtn Cohort 2: 10^8 vp (N=6)	Ad4-H5-Vtn Cohort 3: 10^9 vp (N=9)	Ad4-H5-Vtn Cohort 4: 10^{10} vp (N=6)	Ad4-H5-Vtn Cohort 5: 10^{11} vp (N=4)
Number (%) Seroconverted					
Post-boost	1 (33%)	2 (33%)	4 (44%)	5 (83%)	4 (100%)
GMT (95% CI)					
Post-boost	10 (1,197)	13 (4,44)	14 (5,40)	66 (22,195)	57 (7,468)

Pre-existing Ad4 immunity is defined as a positive result (Ad4 titer > 6) at baseline prior to first vaccination. Post-boost seroconversion requires a 4-fold rise over the last pre-boost value. Percentages and GMTs are calculated from subjects (N) who had a pre-boost and post-boost result.

Table S-14. Number (%) of Subjects Without Pre-existing Ad4 Immunity who Seroconverted by HAI Post-boost and GMT (95% CI)

Study Day	Ad4-H5-Vtn Cohort 1: 10^7 vp (N=10)	Ad4-H5-Vtn Cohort 2: 10^8 vp (N=12)	Ad4-H5-Vtn Cohort 3: 10^9 vp (N=9)	Ad4-H5-Vtn Cohort 4: 10^{10} vp (N=9)	Ad4-H5-Vtn Cohort 5: 10^{11} vp (N=15)
Number (%) Seroconverted					
Post-boost	8 (80%)	12 (100%)	8 (89%)	7 (78%)	15 (100%)
GMT (95% CI)					
Post-boost	38 (15,96)	93 (54,161)	82 (31,220)	84 (28,252)	170 (125,232)

Pre-existing Ad4 immunity is defined as a positive result (Ad4 titer > 6) at baseline prior to first vaccination. Post-boost seroconversion requires a 4-fold rise over the last pre-boost value. Percentages and GMTs are calculated from subjects (N) who had a pre-boost and post-boost result.

References

1. Alexander J, Ward S, Mendy J, et al. Pre-clinical evaluation of a replication-competent recombinant adenovirus serotype 4 vaccine expressing influenza h5 hemagglutinin. PLoS One 2012;7:e31177.
2. Lyons A, Longfield J, Kuschner R, et al. A double-blind, placebo-controlled study of the safety and immunogenicity of live, oral type 4 and type 7 adenovirus vaccines in adults. Vaccine 2008;26:2890-8.
3. Horimoto T, Kawaoka Y. Reverse genetics provides direct evidence for a correlation of hemagglutinin cleavability and virulence of an avian influenza A virus. J Virol 1994;68:3120-8.
4. Shabram PW, Giroux DD, Goudreau AM, et al. Analytical anion-exchange HPLC of recombinant type-5 adenoviral particles. Human gene therapy 1997;8:453-65.
5. WHO Manual on Animal Influenza Diagnosis and Surveillance; 2002.
6. Crawford-Miksza LK, Schnurr DP. Quantitative colorimetric microneutralization assay for characterization of adenoviruses. J Clin Microbiol 1994;32:2331-4.
7. McCullers JA, Van De Velde LA, Allison KJ, Branum KC, Webby RJ, Flynn PM. Recipients of vaccine against the 1976 "swine flu" have enhanced neutralization responses to the 2009 novel H1N1 influenza virus. Clin Infect Dis 2010;50:1487-92.
8. Rowe T, Abernathy RA, Hu-Primmer J, et al. Detection of antibody to avian influenza A (H5N1) virus in human serum by using a combination of serologic assays. J Clin Microbiol 1999;37:937-43.
9. Lewis JJ, Janetzki S, Schaed S, et al. Evaluation of CD8(+) T-cell frequencies by the Elispot assay in healthy individuals and in patients with metastatic melanoma immunized with tyrosinase peptide. Int J Cancer 2000;87:391-8.
10. Currier JR, Kuta EG, Turk E, et al. A panel of MHC class I restricted viral peptides for use as a quality control for vaccine trial ELISPOT assays. J Immunol Methods 2002;260:157-72.
